# Demographic history and selection at HLA loci in Native Americans

**DOI:** 10.1371/journal.pone.0241282

**Published:** 2020-11-04

**Authors:** Richard M. Single, Diogo Meyer, Kelly Nunes, Rodrigo Santos Francisco, Tábita Hünemeier, Martin Maiers, Carolyn K. Hurley, Gabriel Bedoya, Carla Gallo, Ana Magdalena Hurtado, Elena Llop, Maria Luiza Petzl-Erler, Giovanni Poletti, Francisco Rothhammer, Luiza Tsuneto, William Klitz, Andrés Ruiz-Linares

**Affiliations:** 1 Department of Mathematics and Statistics, University of Vermont, Burlington, Vermont, United States of America; 2 Departmento de Genética e Biologia Evolutiva, Universidade de São Paulo, São Paulo, Brazil; 3 Center for International Blood and Marrow Transplant Research, Minneapolis, Minnesota, United States of America; 4 CW Bill Young Marrow Donor Recruitment and Research Program, Georgetown University, Washington, DC, United States of America; 5 Instituto de Biología, Universidad de Antioquia Medellín, Medellín, Colombia; 6 Laboratorios de Investigación y Desarrollo, Universidad Peruana Cayetano Heredia, Lima, Peru; 7 School of Human Evolution and Social Change, Arizona State University, Tempe, Arizona, United States of America; 8 Programa de Genética Humana, Instituto de Ciencias Biomédicas, Facultad de Medicina, Universidad de Chile, Santiago, Chile; 9 Departamento de Genética, Universidade Federal do Paraná, Curitiba, Paraná, Brazil; 10 Facultad de Medicina, Universidad Peruana Cayetano Heredia, Lima, Peru; 11 Instituto de Alta Investigación, Tarapacá University, Arica, Chile; 12 Department of Basic Health Sciences, Universidade Estadual de Maringá, Maringá, Paraná, Brazil; 13 Department of Integrative Biology, University of California, Berkeley, California, United States of America; 14 Ministry of Education Key Laboratory of Contemporary Anthropology and Collaborative Innovation Center of Genetics and Development, School of Life Sciences and Human Phenome Institute, Fudan University, Shanghai, China; 15 CNRS, EFS, ADES, D Aix-Marseille University, Marseille, France; Universidade Nova de Lisboa Instituto de Higiene e Medicina Tropical, PORTUGAL

## Abstract

The American continent was the last to be occupied by modern humans, and native populations bear the marks of recent expansions, bottlenecks, natural selection, and population substructure. Here we investigate how this demographic history has shaped genetic variation at the strongly selected HLA loci. In order to disentangle the relative contributions of selection and demography process, we assembled a dataset with genome-wide microsatellites and *HLA-A*, *-B*, *-C*, and -*DRB1* typing data for a set of 424 Native American individuals. We find that demographic history explains a sizeable fraction of HLA variation, both within and among populations. A striking feature of HLA variation in the Americas is the existence of alleles which are present in the continent but either absent or very rare elsewhere in the world. We show that this feature is consistent with demographic history (i.e., the combination of changes in population size associated with bottlenecks and subsequent population expansions). However, signatures of selection at HLA loci are still visible, with significant evidence selection at deeper timescales for most loci and populations, as well as population differentiation at HLA loci exceeding that seen at neutral markers.

## 1. Introduction

The American continent was the last to be colonized by humans. Most genetic studies corroborate the hypothesis that the first individuals came to America from Northeast Asia, now Siberia, about 15,000 to 18,000 years before present (YBP) through the Beringia land bridge [1–3].

According to this hypothesis, the extant Native American populations are the result of a single migratory wave that entered the American continent at the end of the last glacial period, after a period from 5,000–8,000 YBP in Beringia, which allowed the genetic differentiation of the First Americans [1, 4–6]. After the expansion of this population into the American continent, subsequent waves of migration came to America from Siberia, leaving genetic traces in the current North American populations (e.g., Eskimos and Na-Dene populations) [3, 7–9]. Some studies have also detected a Polynesian genetic component in extinct Native South Americans [10, 11], as well as an Austromelanesian genetic component in contemporary [7] and ancient [12] South Americans. Although the hypothesis of direct contribution of these groups by marine routes was proposed a while ago [13], bioanthropological studies suggest that at the end of the Pleistocene the paleoamerican populations had greater morphological diversity shared with common ancestors of South Asia and Oceania, and that these characteristics may have been retained in some populations or individuals [14, 15].

This recent progress in our knowledge regarding Native American history and demography relies on studies with genomic datasets. These have refined the identification of genetic differences within the American continent, making it possible to cluster the autochthonous populations in major groups, such as Mesoamericans, Andeans, Amazonian and Eskimos [3, 16], and to provide reliable dates and ancestry sources.

While the study of small numbers of autosomal loci is less powerful for testing evolutionary scenarios with large numbers of parameters, specific loci with well-known functional roles can provide insights into the combination of demographic and selective events that shaped the distribution and variation of a specific gene or genomic region [17].

In this study, we compare the patterns of variation and differentiation at HLA loci with a set of putatively neutral markers, in Native American populations. Our primary goal is to provide a deeper understanding of the evolutionary forces that have shaped variation at HLA genes within the American continent. The demographic history of Native Americans, well explored in many studies [3, 18, 19], provides the context within which we analyze variation of three HLA class I genes (*HLA-A*, *-B*, *and -C*) and one HLA class II gene (*HLA-DRB1*).

Previous studies have shown that demographic history and selective forces interact and leave strong signatures in the variation of HLA genes [20–22], but understanding details regarding this interaction remains a challenge. Outstanding questions concern whether demographic effects can override selective signatures and if selection can revert gradients of allele frequencies generated by geography [23]. Native American populations provide a valuable case study to understand the interaction of natural selection and demographic history for several reasons. First, when occupying the American continent, populations encountered novel pathogens, types of food, and climates. Jointly, these factors posed new selective challenges to the populations which reached the continent. Second, the founding of the Americas likely involved an extreme bottleneck, causing a marked reduction in diversity with respect to other world regions [19, 24–27], highlighting the importance of analyzing the relative intensity of selection and drift in shaping extant Native American HLA diversity. Finally, for more than two decades we have known that Native Americans exhibit HLA alleles which are present exclusively in this continent, and are not found in any other region [28, 29], raising the question as to whether this pattern is a consequence of selection favoring locally advantageous alleles or a consequence of intense genetic drift.

In order to disentangle the relative contributions of demography and selection on specific loci of interest it is essential to have a large dataset, which includes markers that document the demographic history of the sampled populations. In the present study we take such an approach, using HLA sequence data and a genome-wide microsatellites dataset from a panel of 424 Native American individuals. We use the microsatellites as a demographic control and evaluate the differences between variation at these neutral markers and that of the HLA genes. Specifically, we examine how well the putatively neutral microsatellites predict variation and differentiation at the strongly selected HLA genes. We also systematically survey the geographic distribution of HLA alleles, with particular emphasis on those that are restricted to the American continent, so as to investigate the relative importance of selection and drift on patterns of geographic variation.

## 2. Materials and methods

### Population sampling

**Fig 1:**
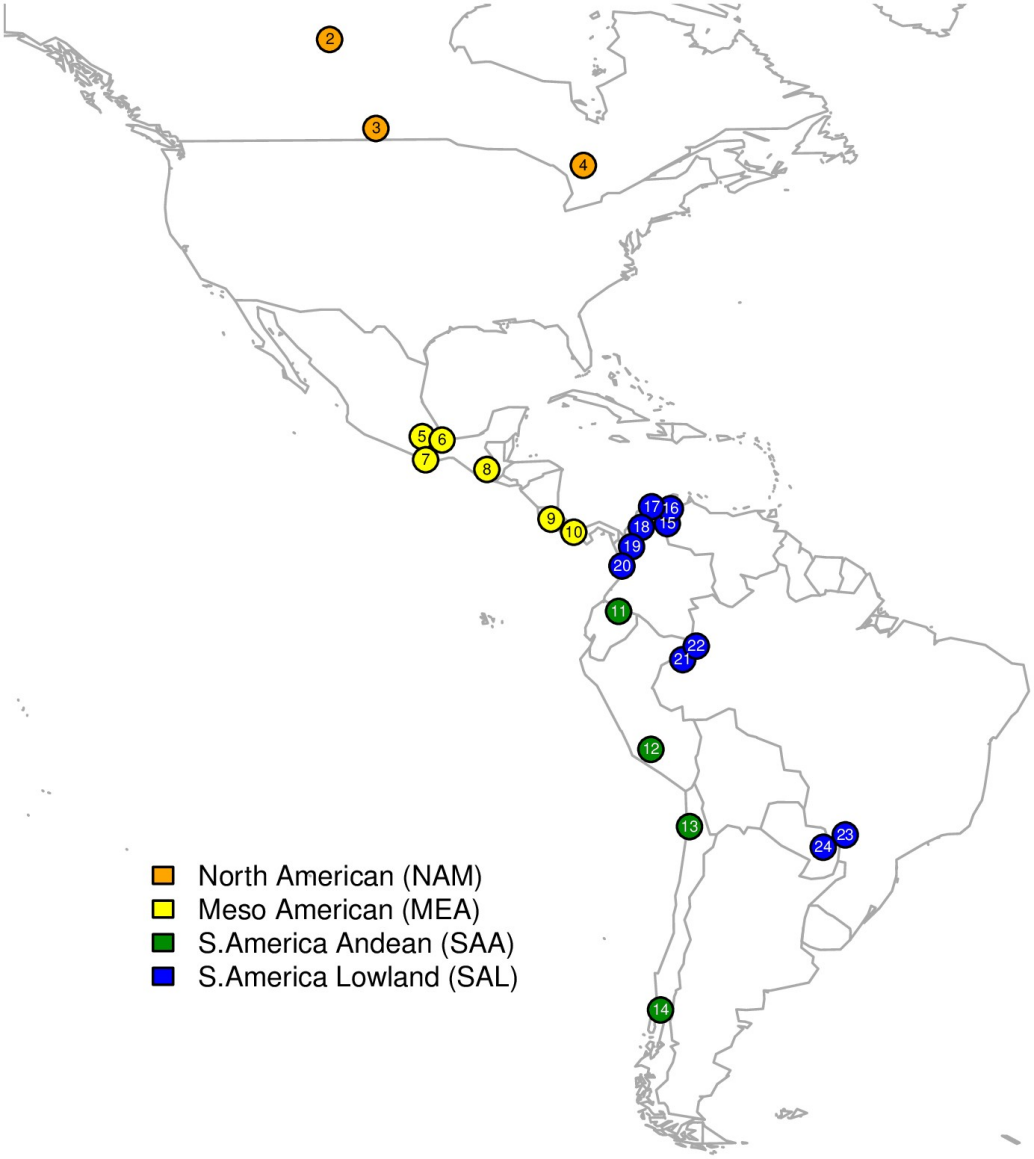
Map of North and South America with location of Native American populations studied. Locations indicated based on geographic coordinates given in Table 1.

We studied microsatellite and HLA variation in 424 individuals from 23 Native American and one Siberian population (Table 1, Fig 1). The Siberian population was not used in analyses concerning diversity and differentiation within the Americas, but provides insight into the differences between the Americas and a population likely related to their close ancestors.

**Table 1:** Sample sizes and heterozygosity.

	Population	Region^a^	lat^b^	lon^c^	No. of chromosomes				Heterozygosity			
					A	B	C	DRB1	A	B	C	DRB1
1	TundraNentsi	SIB	66.1	76.5	32	32	30	32	0.777	0.863	0.860	0.879
2	Chipewyan	NAM	59.6	-107.3	50	50	50	50	0.685	0.888	0.895	0.890
3	Cree	NAM	50.3	-102.5	36	36	36	36	0.793	0.852	0.903	0.903
4	Ojibwa	NAM	46.5	-81	30	30	30	32	0.820	0.871	0.871	0.863
5	Mixtec	MEA	17	-97	40	40	40	40	0.704	0.864	0.716	0.765
6	Mixe	MEA	17	-96	40	40	40	40	0.708	0.809	0.736	0.782
7	Zapotec	MEA	16	-97	40	34	32	36	0.730	0.927	0.797	0.878
8	Kaqchikel	MEA	15	-91	38	38	38	38	0.844	0.922	0.861	0.881
9	Cabecar	MEA	9.5	-84	38	38	38	38	0.770	0.676	0.637	0.709
10	Guaymi	MEA	8.5	-82	36	36	36	36	0.660	0.716	0.619	0.671
11	Inga	SAA	1	-77	30	28	30	30	0.780	0.855	0.773	0.853
12	Quechua	SAA	-14	-74	42	42	42	42	0.531	0.917	0.816	0.832
13	Aymara	SAA	-22	-70	40	40	40	40	0.622	0.816	0.688	0.775
14	Huilliche	SAA	-41	-73	40	40	30	40	0.809	0.778	0.782	0.848
15	Wayuu	SAL	11	-73	30	30	28	30	0.889	0.878	0.865	0.873
16	Arhuaco	SAL	11	-73.8	34	34	34	34	0.740	0.734	0.749	0.742
17	Kogi	SAL	11	-74	30	28	28	28	0.658	0.694	0.617	0.684
18	Zenu	SAL	9	-75	24	32	24	24	0.740	0.834	0.812	0.580
19	Embera	SAL	7	-76	28	28	28	28	0.668	0.755	0.643	0.737
20	Waunana	SAL	5	-77	40	40	40	40	0.560	0.849	0.784	0.691
21	TicunaArara	SAL	-4	-70	30	28	28	30	0.584	0.821	0.730	0.709
22	TicunaTarapaca	SAL	-4	-70	38	38	34	38	0.647	0.749	0.645	0.601
23	Guarani	SAL	-23	-54	20	20	18	18	0.805	0.785	0.759	0.753
24	Ache	SAL	-24	-56	26	22	16	24	0.541	0.550	0.398	0.295

^a^SIB = Siberia, NAM = N.America, MEA = Meso America, SAA = S.America Andes, SAL = S.America Lowlands.

^b^Latitude (degrees North),

^c^Longitude (degrees East).

The populations were divided into five groups based on geographic and linguistic criteria, according to Hünemeier et al. [16] and Reich et al. [3]–North America (NAM), Mesoamerica (MEA), South America Lowland (SAL), the South America Andean (SAA), and Siberian (SIB). A previous in depth study of these populations showed that most completely lacked European or African ancestry, or at most had contributions lower than 5%.

### Microsatellite dataset

We assembled a dataset consisting exclusively of individuals typed for the set of 678 genome-wide microsatellites and also typed for HLA loci (described below). Details about the set of microsatellite markers are described in [18].

### HLA typing

To identify the class I *HLA-A*,*-B*,*-C* alleles carried by each individual, PCR primers were used to amplify each locus as previously described [30]. HLA typing was carried out on DNA samples previously extracted for the study of Wang et al. [18]. Applied Biosystems Big Dye terminator chemistry and sequencing primers were used to obtain the sequences of both strands of exons 2 and 3. Exon 2 of the class II *HLA-DRB1* alleles were amplified and sequenced using the AlleleSEQR class II kit (Abbott Molecular Inc, Des Plaines, IL). Additional in-house PCR and sequencing primers were added when needed to obtain resolution. Reaction products were identified with Applied Biosystems 3730xl DNA analyzer (PE Applied Biosystems, Foster City, CA) and sequence interpretation used Assign software (Conexio Genomics, Applecross, Western Australia).

For subjects with multiple possible class I genotypes, either allele specific sequencing primers or allele specific PCR amplification were used to identify the specific allele combination present. Interpretation of alternative genotypes (i.e. allele pairs) used the IPD-IMGT/HLA Database (database release 2.19.0, October 2007). In-house primer sequences are available at https://genevol.ib.usp.br/software/. Alternative alleles identical in exons 2 and 3 (class I) or exon 2 (*DRB1*) were not resolved. Unresolved alleles of this type that differ in two-field names, i.e., encode allelic products that vary in amino acid sequence outside of the antigen binding site, are indicated by the use of a “g” following the name of the lowest numbered allele in the group. For example, A*02:*01*:*01G* includes alleles A*02:*01*:*01*:*01*, A*02:*09*, A*02:*43N*, A*02:*66* as well as synonymous alleles A*02:*01*:*01*:02L and A*02:*01*:*08*. A listing of these unresolved alleles can be found at http://www.ebi.ac.uk/imgt/hla/ambig.html under database release 2.21.0. A few *DRB1* alleles differing in the last three codons of exon 2 were also not distinguished.

The *HLA-B* alleles of a subset of Native Americans (n = 148) were sequenced in Dr. Meyer’s laboratory as part of another project. After resolution of discrepancies, typing for four samples (2.7%) was corrected.

For most analyses (see below) we transformed the allele calls to two-field allele definitions, where the first field identifies the serological group and the second field defines the peptide sequence, specifying HLA proteins. This results in a reduction in the amount of information about the molecular level definition of the allele’s sequence, but allowed us to compare our dataset with that of previously published work, which is almost exclusively at the two-field level. The mapping between the molecular-level and two-field level of resolution was one-to-one for all but a small number of alleles (*HLA-A*: *24*:*03*:*01G 24*:*03*:*02; HLA-B*: *39*:*06*:*01*,*39*:*06*:*02*, *51*:*13*:*01*, *51*:*13*:*02*, *52*:*01*:*01G*, *52*:*01*:*02; HLA-DRB1*: *04*:*05*:*01*, *04*:*05*:*04*), implying that the information loss in minimal. For a subset of the analyses we used sequence information available from our typing to carry out tests that use this level of information (tests for equilibrium-neutrality that explore the site-frequency spectrum).

The genotype data used in this study are available in S1 Table and at the Allele Frequency Net Database (AFND) repository for immune-related gene polymorphisms in worldwide populations. The AFND accession numbers for the 24 populations in this study are 3692–3715. For example, the TundraNentsi population from Siberia can be accessed at http://www.allelefrequencies.net/population/AFND3692. A listing of the 24 hyperlinked accession URLs can be found at https://genevol.ib.usp.br/software/.

### Ethics statement

The samples analysed here were collected as part of a previous research project [3] with informed consent encompassing genetic studies of population history. Institutional approval in the country of collection was obtained for the use of each set of samples in such research. Ethical oversight and approval for this project was provided by the National Health Service National Research Ethics Service, Central London committee (reference no. 05/Q0505/31).

### Data analyses

#### Population variability

We used the PyPop (v.0.7.0) software package [31, 32] to estimate summary statistics (the number of alleles (k), and sample heterozygosity (H)) and to test for deviation from Hardy-Weinberg proportions (HWP) using an exact test [33].

#### Tests of neutrality

We tested for departure from neutrality-equilibrium conditions using two methods, which capture different aspects of the time scale for selection. We used the Ewens-Watterson test [34, 35] implemented in PyPop to test for deviations from neutrality in the direction of balancing selection. The full molecular-level dataset was analyzed using Tajima’s-D, as implemented in Arlequin (v.3.5) [36]. For each method deviations in the direction of positive selection are indicated with p-values close to one.

#### Differentiation among populations

Differentiation among pairs of populations was estimated using AMOVA, as implemented in the ADE4 (v.1.6–2) package of R to compute F_ST_ values. Pairwise estimates were then used to estimate mean differentiation among populations within a region, and among populations from different pairs of regions.

We developed an empirical approach to place the *F*_*ST*_ results for HLA loci in the context of differentiation of putatively neutral microsatellites. For each pair of populations, each HLA *F*_*ST*_ result was normalized by subtracting the mean *F*_*ST*_ for the 678 microsatellites and then dividing by the standard deviation of the *F*_*ST*_ s for the microsatellites:

$$Z_{ST}=[F_{ST\_HLA}-mean(F_{ST}{\_}_{msats})]/SD(F_{ST}{\_}_{msats}).$$

This measure summarizes how many standard deviations the HLA *F*_*ST*_ values deviates from that of the neutral loci. An empirical p-value was computed as the proportion of the 678 microsatellite loci with a higher *F*_*ST*_ than HLA.

#### Haplotype frequencies

Haplotype frequencies were estimated using the EM algorithm [37] as implemented in PyPop.

#### Allele sharing between the Americas and other world regions

For each allele in our dataset (at the two-field or peptide level of resolution), we compared the mean frequency across the Native American populations with the mean frequency from 364 non-Native American populations from a published meta-analysis of 497 worldwide populations [21] representing approximately 66,800 individuals. These mean frequencies, computed after omitting migrant populations and those whose continental origin was classification was uncertain, were used to estimate a ratio of frequencies between the Americas and other world regions. We assigned alleles to one of three categories:

1. *endemic alleles*: those present only in the Americas (and completely absent in all other regions);
2. *large frequency differences (LFD) alleles*: those present both in the Americas and other world regions, but at least 3-fold more common in the Americas with respect to other regions were referred to as *large frequency difference (LFD) alleles*;
3. *Other*: non-endemic, non-LFD alleles.

We next applied a set of filters to this classification. First, if an endemic or LFD allele was present in 3 or fewer copies in the Americas, we considered it "poorly supported". This category contains alleles that were rare in both the Americas and other world regions, but yielded large ratios due to small sample sizes and large sampling variances for frequencies in the Americas. Second, we performed a similar classification comparing allele frequencies in the Native American and non-Native American populations of the Solberg et al. [21] study, so as to validate the findings obtained from our dataset. If an LFD allele was poorly supported in both datasets, we removed it from our final set of classified alleles (21 LFD alleles were poorly supported– 3 *HLA-A*, 13 *HLA-B*, and 5 *HLA-DRB1*). This yielded a final list of alleles found in our Native American data which were classified as either endemic or LFD.

Because the HLA typing for the Solberg et al. [21] dataset was carried out over an extended period of time, it is possible that the same allele could have received different names, depending on when it was typed, since earlier methods may not have been able to unambiguously distinguish among certain alleles. A consequence of this naming inconsistency is the possibility that a subset of alleles we classify as endemic are in fact shared among regions, but have been given different names across studies. To minimize the possibility that changes in allele names had any impact on our analyses, we used an empirical approach to quantify the degree of ambiguity in allele calling, and we report this information in S2 Table.

## 3. Results and discussion

### 3.1 HLA alleles within the Americas

For the 23 Native American populations and one Siberian we identified 36 alleles at *HLA-A*, 80 at *HLA-B*, 29 at *HLA-C*, and 38 at *DRB1*, at the two-field level of resolution (S1 Table). This dataset was used in all analyses, with the exception of neutrality tests, which were carried out on a version of the data which was recoded at the molecular level (by assigning DNA sequences corresponding to each allele).

### 3.2 Deviation from HWP

Out of the total of 96 tests for deviation from HWP (23 Native Americans and one Siberian population, at 4 loci), five were significant at the 0.05 level of significance, which is close to that expected by chance alone (4.8 tests). The deviations are spread over all loci (one in each and two in *HLA-B*) and occur in five different populations (HLA-A: Zapotec, HLA-B: Kogi and TicunaTarapaca, HLA-DRB1: Waunana). In each case small, but significant, overrepresentation of specific individual genotypes contributed to the overall locus-level deviation (A*02:06+A*31:*01*, B*35:43+B*40:*02*, C*04:01+C*05:*01*, DRB1*04:07+DRB1*04:*11*, DRB1*04:04+DRB1*14:*02*). Deviations are due to an excess of heterozygotes, arguing against the presence of null alleles or allele dropouts. The observed deviations were small and non-significant when we account for multiple testing, and thus differ from a classic finding for Native Americans which identified HWP deviations consistent with heterozygote advantage [38]. No population or locus was consistently overrepresented among the deviations, arguing against the possibility that demographic or technical factors account for deviations.

### 3.3 Geographic variation in heterozygosity

**Fig 2:**
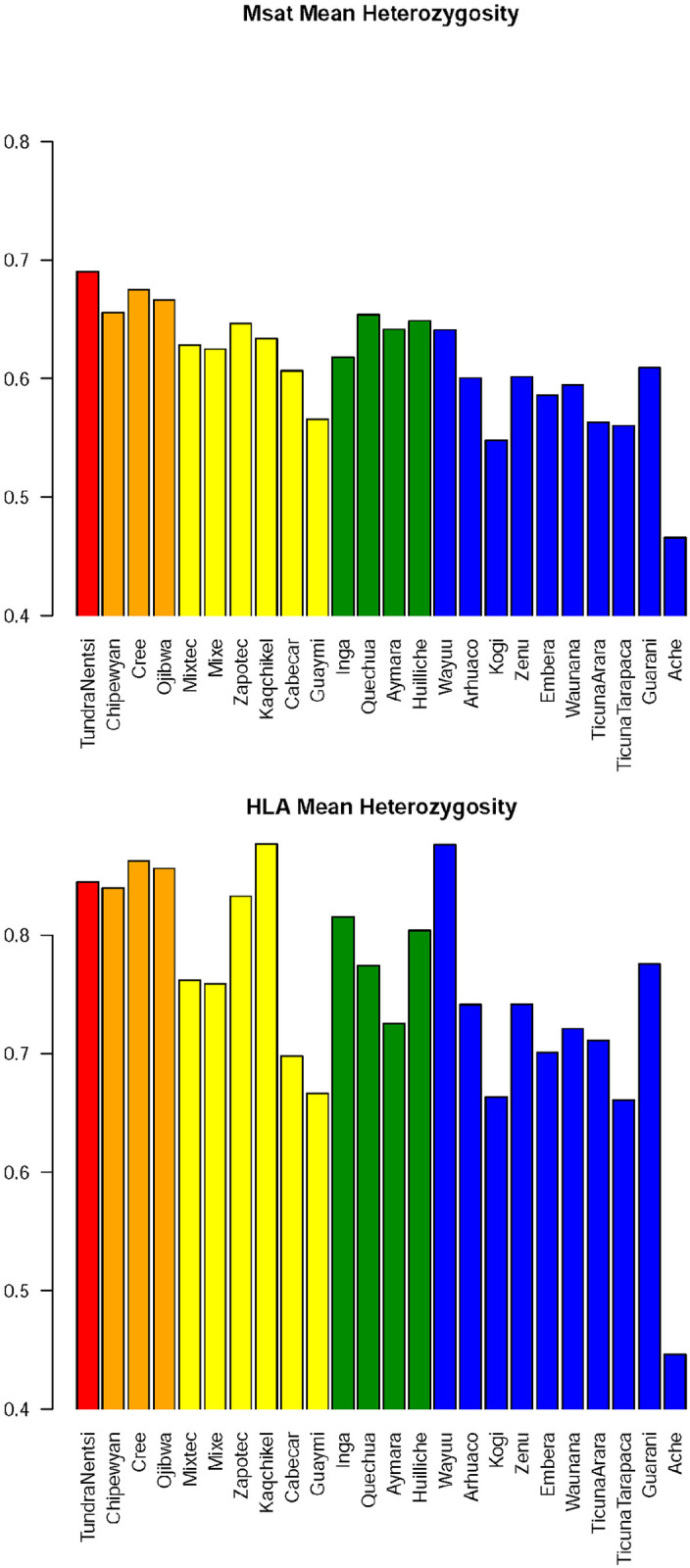
(a, b) Heterozygosity values per population and geographic region (a) Microsatellites, (b) HLA. Populations are ordered within geographic regions based on the least cost path (Wang et al, 2007), which indicates the distance from Siberia along likely migration routes.

We examined the degree to which demographic history accounts for differences in heterozygosities among populations and regions of the Americas. We ordered populations within geographic regions based on the least cost path (as reported by [18]), which measures how far each population travelled from a putative Siberian source, providing a proxy for the amount of drift experienced. For the microsatellites there is a general decrease of heterozygosity, with the South American Lowland populations (SAL) having lowest heterozygosities, Meso Americans (MEA) showing slightly larger values, followed by the Andeans (SAA) (Fig 2a). North Americans (NAM) have higher heterozygosities than all South Americans. These differences are supported statistically, with pairwise contrasts in heterozygosity being greatest in contrasts between NAM-MEA (p = 0.067), SAL-NAM (p = 0.001), SAL-SAA (p = 0.017, tests controlled for multiple comparisons at the 0.05 level using Tukey’s HSD method; we conservatively excluded the Aché to avoid effects driven by this one extreme population, cf. [18]).

The variation in HLA genes among regions does not show a strong relationship with distance. Only the SAL-NAM contrast in heterozygosity was significant at the 0.05 level for all loci (p < 0.05 for all loci combined; Fig 2b).

**Fig 3:**
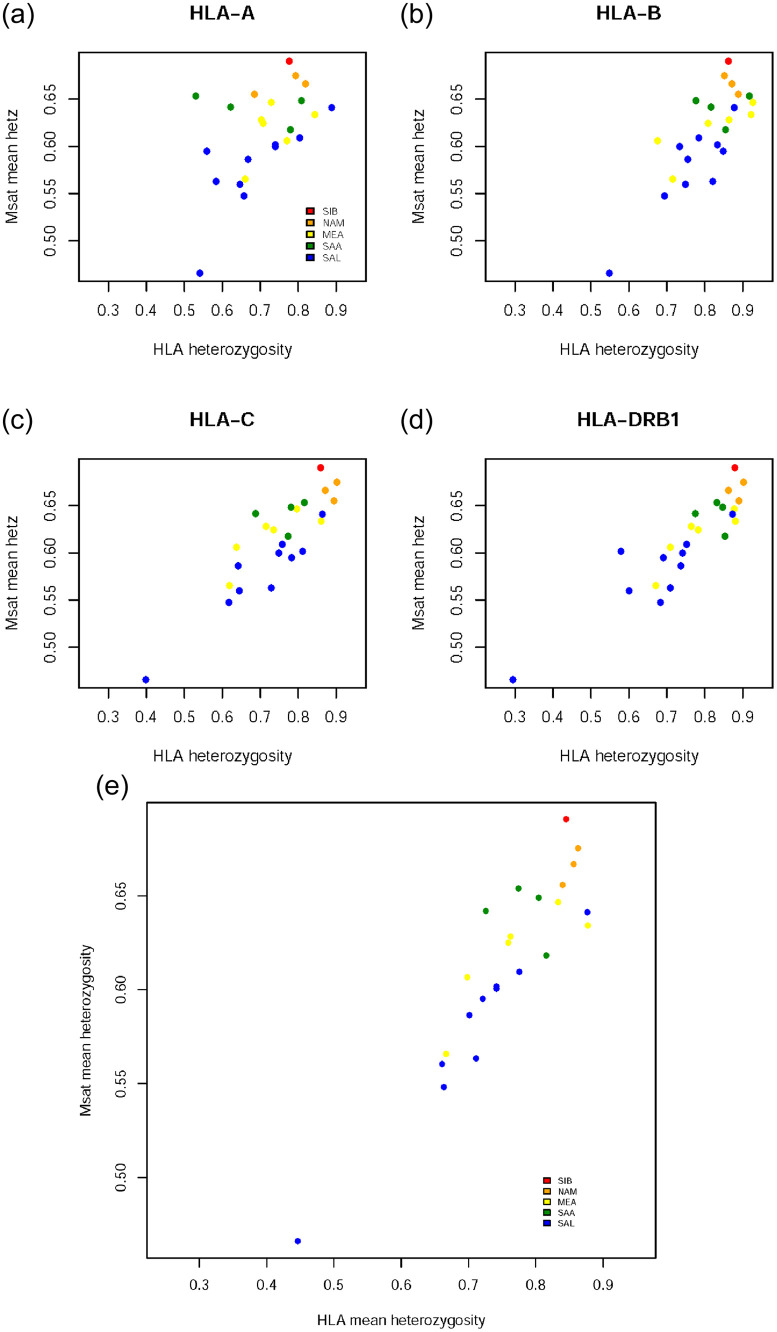
(a–d) Relationship between heterozygosity for microsatellites and HLA. Results are shown separately for each HLA locus in panels (a)-(d). Panel (e) shows results that are averaged over the four HLA loci.

Under the assumption that demographic forces shape variation throughout the entire genome, we expect a correlation in measures of diversity at different loci. We tested this by comparing heterozygosity between HLA loci and the microsatellites (Fig 3, Table 2). All HLA loci have heterozygosities which are highly correlated with that of microsatellites (p<0.01 for all contrasts), and the average heterozygosity for the 4 HLA loci combined has a correlation of r = 0.91 (p<0.01, Pearson correlation) with that of microsatellites (Fig 3e). Fig 3 also allows investigation of microsatellite diversity while conditioning on HLA heterozygosity. Taking a vertical slice of Fig 3 (e.g., HLA heterozygosity between 0.8 and 0.85) one finds lower microsatellite heterozygosity in SAL followed by MEA populations and higher microsatellite heterozygosity in SAA followed by NAM populations. This trend is consistent across populations with different ranges of HLA heterozygosity (i.e., different vertical slices) indicating that trends due to recent demographic history which are visible in microsatellites are also visible regardless of the level of HLA diversity.

**Table 2:** Correlation between mean microsatellite heterozygosity and HLA diversity (heterozygosity and abundance of endemic alleles).

	Heterozygosity		LFD and endemic frequency
	Correlation	p-value	Correlation
HLA-A	0.541	<.01	-0.66
HLA-B	0.798	<.01	-0.58
HLA-C	0.873	<.01	-0.05
HLA-DRB1	0.904	<.01	-0.49

Correlation with locus heterozygosity is presented in the first column and with total frequency of endemic and LFD alleles in the second column. Correlations are over all Native American populations.

These results underscore the fact that demographic processes overwhelm the idiosyncratic selective regimes which affect each locus individually. Thus, although HLA loci have unusually high heterozygosity as a consequence of balancing selection, the signatures of recent demographic history are clearly visible in differences of polymorphism levels among populations.

#### Individual-based levels of heterozygosity

The availability of HLA and microsatellite data for a matched set of individuals allows us to also examine correlations in diversity defined at the level of individuals, in addition to the population level. In order to do this, we quantified the proportion (or absolute number, for HLA) of loci within an individual which are heterozygous and determined a correlation with microsatellite heterozygosity.

**Fig 4:**
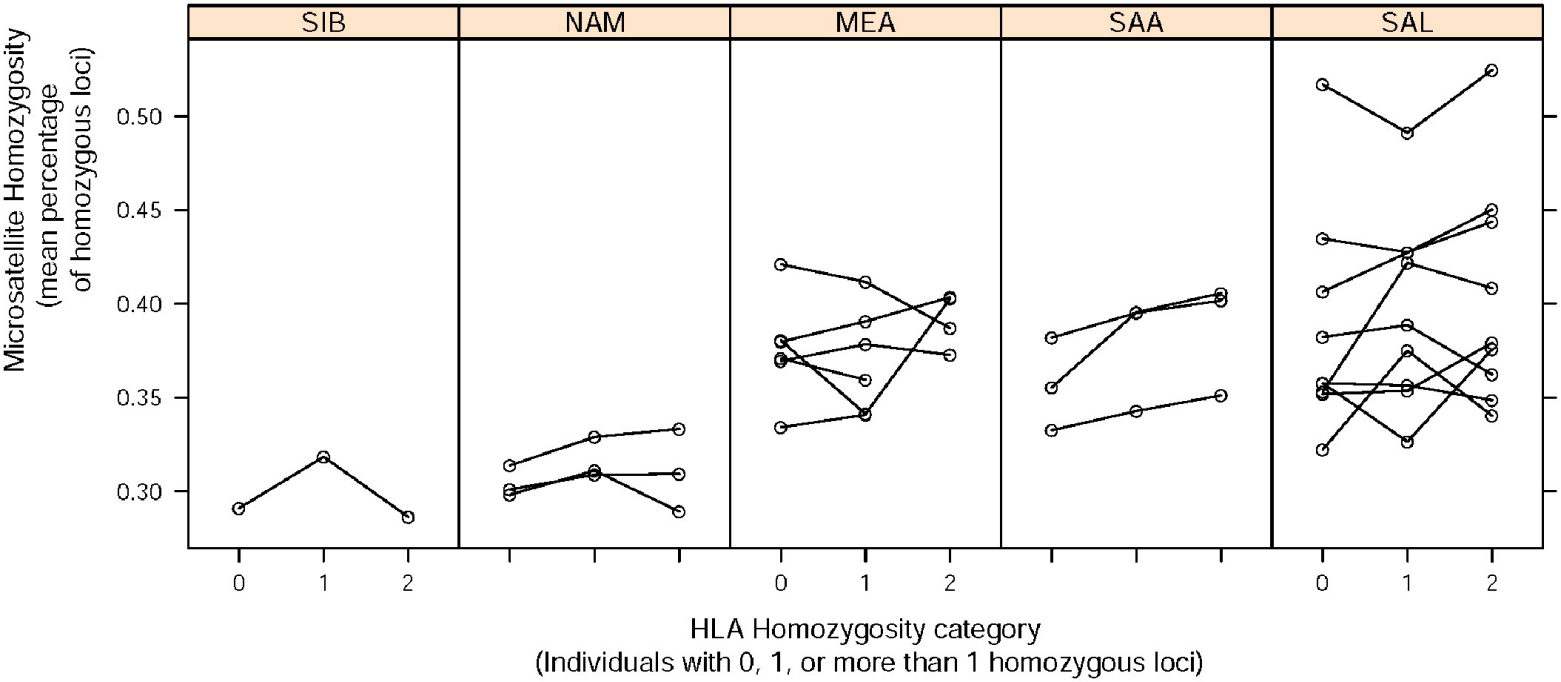
Relationship between the proportion of homozygous loci at HLA and homozygosity at microsatellites averaged over individuals. Each line represents the trend in a specific population.

For each population, the relationship between mean individual homozygosity at HLA and microsatellites is shown in Fig 4, which presents a conditional view of microsatellite variability based on categories of HLA heterozygosity at the individual level. The correlation among the proportion of heterozygous loci for HLA and non-HLA markers is low, with most populations showing a minimal increase in genome-wide proportion of homozygous loci along with the equivalent measure at HLA. Of the 22 populations tested, only 3 showed a significant association between HLA and microsatellite homozygosity, after correction for multiple testing (Ticuna Tarapaca, Huilliche, Embera; p < 0.0025).

**Fig 5:**
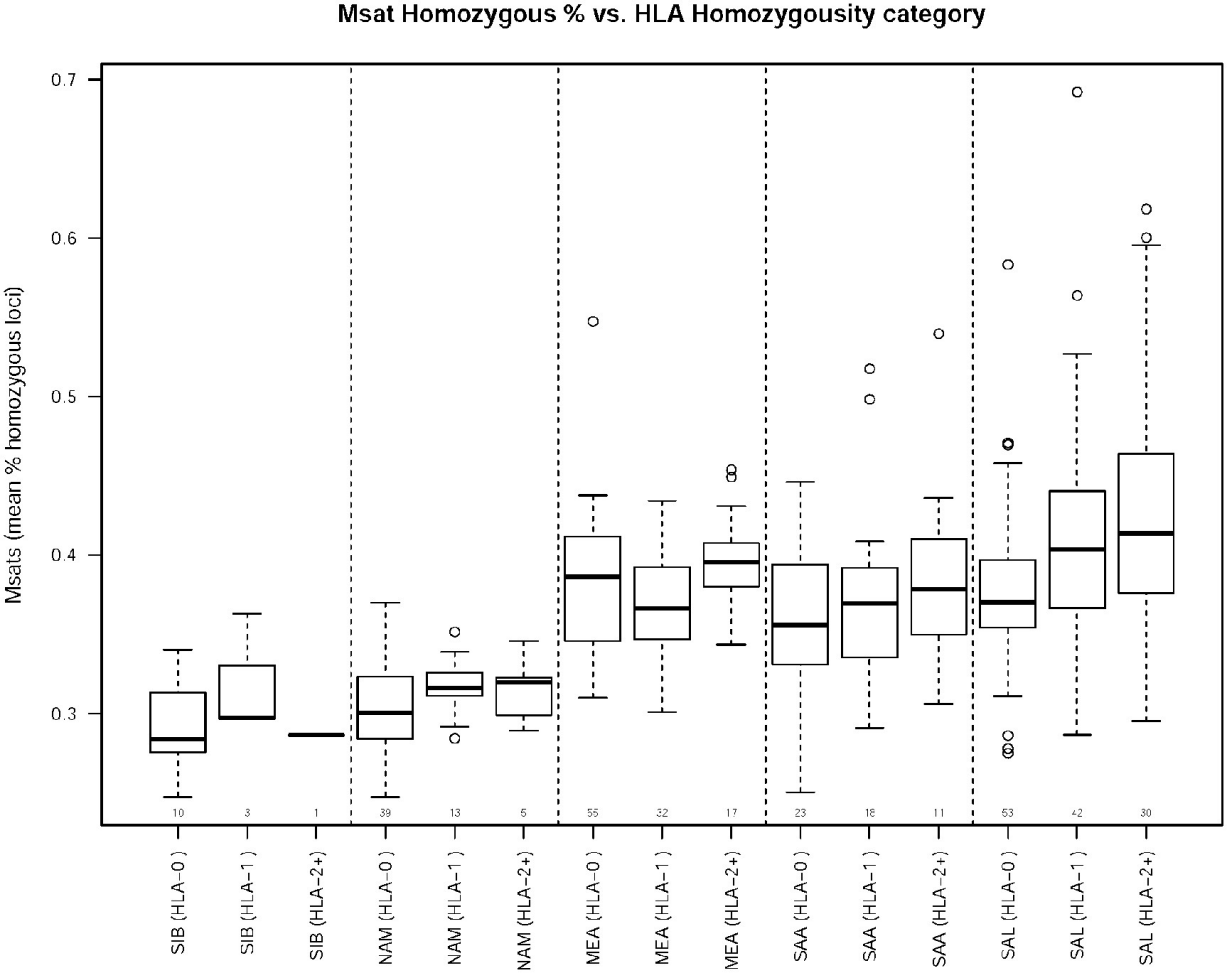
Relationship between zygosity at HLA loci and the average proportion of microsatellite loci for which individuals are homozygous. The proportion of microsatellite loci that are homozygous in each individual is summarized over individuals from each geographic region, separately for individuals with zero, one, or at least two homozygous HLA loci. The number of individuals in the geographic region contributing to each boxplot is shown on the horizontal axis.

Another way to assess the relationship between microsatellite and HLA diversity is to compare the proportion of microsatellite loci which are homozygous for different groups of individuals: (0) those heterozygous for all HLA loci, (1) those homozygous at one HLA locus, and (2) those homozygous for two or more HLA loci (Fig 5). Only one of the regions (SAL, p<0.001) had a significant increasing trend in microsatellite homozygosity for increasing numbers of HLA homozygous loci. Overall, these results show that being heterozygous at HLA loci is not a strong predictor for heterozygosity of the microsatellite loci at the within-individual level.

At first sight these results might appear to contradict the finding of a strong correlation between HLA and microsatellite heterozygosity (Fig 3). However, this discrepancy can be understood by considering that the correlation among HLA and microsatellite variation is explained by overall differences in demographic histories among the populations and not by the inter-individual differences in heterozygosity. Thus, while differences in the history among populations shape HLA and microsatellite variation, within each population (i.e., among individuals) variation is largely stochastic and there are few genome-wide systematic differences in diversity among HLA homozygous and heterozygous individuals. This underscores the importance of population history in shaping variation at both neutral and HLA markers.

### 3.4 Deviation from equilibrium-neutrality expectations

Although there is little controversy regarding the importance of balancing selection in shaping variability at HLA loci [20, 21, 39, 40], it is not clear how evidence for selection varies among populations and what the timescale is for selection on the HLA loci. We addressed these issues by applying two tests for selection to the HLA data. We first tested for deviations from the infinite alleles model using the Ewens-Watterson test (Table 3). We found weak evidence of selection, with the only significant deviations in the direction of balancing selection (*HLA-B* in Chipewyan, *DRB1* in Wayuu, and *HLA-C* in Chipewyan and Zapotec). As was the case for tests of deviation from HWP, the number of significant results approaches that expected for a given level of significance.

**Table 3:** Ewens-Watterson neutrality test^a^.

Population		HLA-A				HLA-B				HLA-C			
		n	Obs F	Exp F	p-value	n	Obs F	Exp F	p-value	n	Obs F	Exp F	p-value
TundraNentsi	SIB	32	0.22	0.19	0.771	32	0.14	0.15	0.423	30	0.14	0.13	0.708
Chipewyan	NAM	50	0.32	0.33	0.557	50	***0*.*11***	***0*.*18***	***0*.*014***	50	***0*.*10***	***0*.*18***	***0*.*001***
Cree	NAM	36	0.21	0.23	0.465	36	0.15	0.12	0.901	36	0.10	0.12	0.217
Ojibwa	NAM	30	0.18	0.24	0.160	30	0.13	0.13	0.576	30	0.13	0.13	0.589
Mixtec	MEA	40	0.30	0.30	0.568	40	0.14	0.15	0.443	40	0.28	0.27	0.673
Mixe	MEA	40	0.29	0.42	0.143	40	0.19	0.21	0.475	40	0.26	0.31	0.392
Zapotec	MEA	40	0.27	0.26	0.631	34	0.07	0.09	0.126	32	***0*.*20***	***0*.*34***	***0*.*021***
Kaqchikel	MEA	38	0.16	0.13	0.807	38	0.08	0.08	0.553	38	0.14	0.13	0.693
Cabecar	MEA	38	0.23	0.30	0.202	38	0.32	0.35	0.510	38	0.36	0.36	0.614
Guaymi	MEA	36	0.34	0.35	0.564	36	0.28	0.30	0.552	36	0.38	0.60	0.056
Inga	SAA	30	0.22	0.21	0.658	28	0.15	0.13	0.830	30	0.23	0.19	0.831
Quechua	SAA	42	***0*.*47***	***0*.*21***	***0*.*997***	42	0.08	0.09	0.385	42	0.18	0.21	0.406
Aymara	SAA	40	0.38	0.31	0.808	40	0.18	0.14	0.927	40	0.31	0.19	0.970
Huilliche	SAA	40	0.19	0.19	0.638	40	***0*.*22***	***0*.*14***	***0*.*975***	30	0.22	0.18	0.826
Wayuu	SAL	30	0.11	0.13	0.256	30	0.12	0.13	0.487	28	0.14	0.16	0.282
Arhuaco	SAL	34	0.26	0.26	0.623	34	0.27	0.25	0.651	34	0.25	0.34	0.184
Kogi	SAL	30	0.34	0.39	0.417	28	0.31	0.33	0.489	28	0.38	0.27	0.910
Zenu	SAL	24	0.26	0.31	0.316	32	0.17	0.19	0.377	24	0.19	0.26	0.088
Embera	SAL	28	0.33	0.28	0.806	28	0.24	0.24	0.668	28	0.36	0.38	0.504
Waunana	SAL	40	0.44	0.36	0.804	40	0.15	0.18	0.253	40	0.22	0.31	0.132
TicunaArara	SAL	30	0.42	0.48	0.410	28	0.18	0.18	0.588	28	0.27	0.28	0.563
TicunaTarapaca	SAL	38	0.35	0.42	0.372	38	0.25	0.23	0.728	34	0.35	0.34	0.622
Guarani	SAL	20	0.20	0.21	0.523	20	0.21	0.18	0.817	18	0.24	0.28	0.337
Ache	SAL	26	0.46	0.46	0.602	22	0.45	0.37	0.825	16	0.60	0.53	0.766

^a^Entries in bold italics are significant at the 0.05 level for a two-tailed test against the alternative of either balancing or directional selection, with p-values less than 0.025 or greater than 0.975, respectively.

The levels of deviation from neutrality at the allele level found in the present study are substantially lower than for other world regions, where up to 20% of populations deviate from neutrality-equilibrium [21]. This suggests that the greater strength of drift in the recent history of Native Americans, which is reflected in the decreased polymorphism with respect to other world regions [18], has resulted in a weaker signature of recent allele-level selection at the HLA loci. In this sense, if we take into account that the Native American populations have undergone a substantial bottleneck in the last 500 years (post-contact) [41], it is expected that the demographic signal is more intense than the selective one.

The finding that HLA variability is well accounted for by demographic history, as captured by the microsatellite data, is not incompatible with a role of selection on HLA loci at a deeper timescale [42]. To examine this possibility, we analyzed the HLA data after recoding it in the form of molecular level variation (i.e., by transforming each allele call into a DNA sequence), and using a method which is sensitive to selective and demographic history on a deeper timescale [43]. The *DRB1* locus was not included since the typing resolution did not allow unique assignments of sequences to each allele. In strong contrast to the findings for the Ewens-Watterson (EW) test, for *HLA-A*, *-B* and *-C* alleles defined at the sequence level, we found positive and significant deviations from the neutrality-equilibrium expectation in 8, 5 and 10 of the Native American populations, respectively (Table 4), corresponding to 35%, 22% and 43% of the 23 sampled populations with a p-value < 0.05 for a one sided test with an alternative hypothesis of balancing selection. These results confirm earlier findings which showed that tests using sequence-level data are substantially more powerful in detecting balancing selection at HLA loci than tests based on the infinite-alleles model (e.g., [39]). We also found a strong trend in the sign of D values: over all loci, at least 21 of the 23 populations show D>0. This overall trend is conservative with expectations under neutrality, since genome-wide there is a shift to negative values of Tajima’s D, likely a consequence of population expansions [2, 19].

**Table 4:** Tajima’s D neutrality test for molecular-level data^a^.

Population		*HLA-A*			*HLA-B*			*HLA-C*		
		n	D	p value	n	D	p value	n	D	p value
TundraNentsi	SIB	32	1.00	0.127	32	1.57	0.038	30	0.73	0.175
Chipewyan	NAM	50	0.95	0.112	50	***2*.*59***	***0*.*003***	50	***1*.*74***	***0*.*032***
Cree	NAM	36	1.22	0.089	36	***1*.*71***	***0*.*025***	36	1.31	0.062
Ojibwa	NAM	30	1.45	0.051	30	1.34	0.065	30	0.91	0.133
Mixtec	MEA	40	1.20	0.082	40	0.91	0.126	40	**1.95**	**0.019**
Mixe	MEA	40	0.32	0.299	40	0.52	0.230	40	***2*.*56***	***0*.*001***
Zapotec	MEA	40	0.20	0.344	34	1.12	0.101	32	***2*.*63***	***0*.*000***
Kaqchikel	MEA	38	***1*.*62***	***0*.*044***	38	***-2*.*17***	***0*.*998***	38	0.77	0.161
Cabecar	MEA	38	***2*.*29***	***0*.*006***	38	0.55	0.226	38	0.69	0.176
Guaymi	MEA	36	1.28	0.074	36	0.03	0.444	36	***2*.*66***	***0*.*001***
Inga	SAA	30	1.18	0.094	28	0.43	0.255	30	1.19	0.087
Quechua	SAA	42	-0.24	0.547	42	0.85	0.147	42	***1*.*46***	***0*.*047***
Aymara	SAA	40	0.66	0.199	40	0.52	0.249	40	0.70	0.171
Huilliche	SAA	40	1.11	0.084	40	1.08	0.097	30	0.73	0.189
Wayuu	SAL	30	***1*.*45***	***0*.*034***	30	1.01	0.116	28	1.29	0.060
Arhuaco	SAL	34	***1*.*99***	***0*.*015***	34	-0.54	0.663	34	***1*.*90***	***0*.*019***
Kogi	SAL	30	***1*.*54***	***0*.*042***	28	0.53	0.237	28	0.77	0.169
Zenu	SAL	24	***2*.*14***	***0*.*002***	32	1.25	0.086	24	***1*.*56***	***0*.*037***
Embera	SAL	28	0.40	0.265	28	1.04	0.106	28	***1*.*94***	***0*.*015***
Waunana	SAL	40	1.02	0.093	40	***2*.*09***	***0*.*012***	40	***1*.*57***	***0*.*033***
TicunaArara	SAL	30	**2.27**	**0.007**	28	***1*.*53***	***0*.*035***	28	1.32	0.075
TicunaTarapaca	SAL	38	***2*.*48***	***0*.*003***	38	***1*.*70***	***0*.*017***	34	1.04	0.125
Guarani	SAL	20	0.96	0.121	20	1.14	0.075	18	-0.10	0.485
Ache	SAL	26	0.73	0.184	22	0.41	0.318	16	0.08	0.440

^a^Entries in bold italics are significant at the 0.05 level for a two-tailed test against the alternative of either balancing or directional selection, with p-values less than 0.025 or greater than 0.975, respectively.

This can be explained by the fact that tests based on the site frequency spectrum, such as Tajima’s D, document the cumulative effect of balancing selection acting on much longer timescales than the EW test, and is thus more powerful [42].

### 3.5 Population differentiation

Adaptation of populations to their local environments is expected to favor alleles that are advantageous in that specific context, and thus drive an increase in the degree of differentiation at the selected loci [44]. On the other hand, models of balancing selection via heterozygote advantage show that genetic differentiation among populations under balancing selection is expected to be decreased with respect to neutral expectations, since this selective regime slows down the rate of genetic drift [45].

To better understand the patterns of genetic differentiation at HLA loci in the Americas we used two approaches. First, we catalogued alleles with large frequency differences between the Americas and other regions. Second, we examined how the degree of interpopulation differentiation at HLA differs from that seen at putatively neutral microsatellite loci.

#### Endemic alleles in the Americas

In order to catalog alleles with marked geographic discontinuities, we compared HLA allele frequencies in the Native American dataset to those from a large meta-analysis [21].

Endemic alleles are present in appreciable number in our dataset: 8, 15, and 5 alleles were endemic for *HLA-A*, *B* and *-C*, respectively (Table 5). However, these alleles contribute relatively little to the total frequency in each population, with mean values per region typically below 2% (Table 6). The exception is *HLA-B*, for which endemic alleles have a mean value of 8% in the Americas, and close to 20% in specific Central and South American populations. This differs from the original reports of endemic alleles [28, 46], according to which the total frequency of endemic alleles was much higher. Since then, many alleles that were previously assumed to be endemic have been found outside the Americas, often at low frequencies.

**Table 5:** Alleles classified as endemic or "large frequency difference" (LFD).

Locus	Endemic^a^	LFD^a^
*HLA-A*	02:64, 31:15, 68:05, 68:16, 68:17, 68:23, 68:30, 68:47	02:06, 02:11, 02:13, 02:17, 02:22, 24:03, 25:01, 31:01, 68:01, 68:03, ***02*:*04*, *02*:*19***
*HLA-B*	15:04, 15:30, 35:19, 35:23, 35:24, 35:48, 35:49, 35:99, 35:102, 39:08, 39:11, 39:19, 40:27, 40:64, 51:13	15:07, 15:08, 27:05, 35:04, 35:05, 35:06, 35:09, 35:10, 35:11, 35:12, 35:14, 35:17, 35:20, 35:21, 35:43, 39:02, 39:03, 39:05, 39:06, 39:09, 40:04, 48:02, 51:08, 51:10, ***15*:*20*, *27*:*03*, *40*:*05*, *48*:*03*, *51*:*02*, *51*:*04***
*HLA-C*	02:07, 03:57, 06:16, 15:09, 15:10	03:05, 04:04, ***15*:*03***
*-DRB1*	08:07	03:02, 04:04, 04:07, 04:11, 08:02, 08:11, 14:06, 14:13, 16:02, ***04*:*17***

^a^The classification procedure is described in the methods section. Alleles in bold italics are found only in the Solberg data, and not in the 424 Native American individuals studied here.

**Table 6:** Sum of frequencies for alleles in endemic and LFD categories.

Population	Region	Number of *Endemic* Alleles				Sum of *Endemic* Allele Frequencies				Sum of *LFD* Allele Frequencies			
		A	B	C	DRB1	A	B	C	DRB1	A	B	C	DRB1
Chipewyan	NAM					0.000	0.000	0.000	0.000	0.160	0.140	0.040	0.100
Cree	NAM			1^c1^		0.000	0.000	0.028	0.000	0.278	0.111	0.028	0.361
Ojibwa	NAM					0.000	0.000	0.000	0.000	0.367	0.067	0.200	0.469
Mixe	MEA					0.000	0.000	0.000	0.000	0.575	0.675	0.025	0.925
Mixtec	MEA		1^b1^	1^c2^		0.000	0.100	0.025	0.000	0.675	0.550	0.025	0.925
Zapotec	MEA		4^b2^^–^^b5^			0.000	0.176	0.000	0.000	0.425	0.500	0.000	0.723
Kaqchikel	MEA	1^a1^	3^b4^^,^^b6^^,^^b7^	1^c3^		0.026	0.078	0.026	0.000	0.237	0.368	0.026	0.579
Cabecar	MEA	1^a2^				0.079	0.000	0.000	0.000	0.552	0.211	0.500	0.763
Guaymi	MEA		2^b8^^,^^b9^			0.000	0.056	0.000	0.000	0.583	0.278	0.167	0.527
Inga	SAA					0.000	0.000	0.000	0.000	0.399	0.251	0.000	0.666
Quechua	SAA	1^a3^	2^b10^^,^^b11^			0.024	0.191	0.000	0.000	0.072	0.334	0.000	0.405
Aymara	SAA	1^a4^	2^b7^^,^^b12^	1^c4^		0.100	0.075	0.025	0.000	0.100	0.475	0.000	0.625
Huilliche	SAA	3^a4^^–^^a6^				0.075	0.000	0.000	0.000	0.375	0.500	0.000	0.575
Kogi	SAL					0.000	0.000	0.000	0.000	0.533	0.465	0.214	0.607
Arhuaco	SAL		1^b13^			0.000	0.176	0.000	0.000	0.441	0.558	0.235	0.646
Wayuu	SAL		1^b8^			0.000	0.100	0.000	0.000	0.533	0.366	0.143	0.667
Zenu	SAL		2^b8^^,^^b14^			0.000	0.187	0.000	0.000	0.583	0.438	0.083	0.875
Embera	SAL		1^b15^	1^c5^		0.000	0.071	0.036	0.000	0.428	0.786	0.000	0.786
Waunana	SAL		1^b10^			0.000	0.150	0.000	0.000	0.350	0.450	0.000	0.475
TicunaArara	SAL		1^b10^			0.000	0.036	0.000	0.000	0.500	0.572	0.036	0.900
TicunaTarapaca	SAL		1^b10^			0.000	0.079	0.000	0.000	0.395	0.395	0.000	0.947
Guarani	SAL	1^a7^	1^b10^			0.050	0.350	0.000	0.000	0.300	0.350	0.000	0.445
Ache	SAL				1^d^	0.000	0.000	0.000	0.083	0.884	0.863	0.000	0.875

^a1^ A*68:05,

^a2^ A*68:30,

^a3^ A*02:64,

^a4^ A*68:17,

^a5^ A*68:16,

^a6^ A*68:23,

^a7^ A*68:47,

^a8^ A*31:15,

^b1^ B*35:23,

^b2^ B*15:30,

^b3^ B*35:24,

^b4^ B*39:08,

^b5^ B*40:27,

^b6^ B*35:48,

^b7^ B*51:13,

^b8^ B*35:49,

^b9^ B*35:102,

^b10^ B*15:04,

^b11^ B*40:64,

^b12^ B*35:19,

^b13^ B*35:99,

^b14^ B*39:19,

^b15^ B*39:11,

^c1^ C*06:16,

^c2^ C*15:09,

^c3^ C*02:07,

^c4^ C*03:57,

^c5^ C*15:10,

^d^ DR*08:07.

Alleles with large frequency differences (LFD) on the other hand, make up a substantial proportion of Native American alleles at all loci, with the exception of *HLA-C* (Table 6). For *HLA-A*, the mean frequency of all LFD alleles taken together is 50% in MEA and 45% in SAL. For *HLA-B*, MEA, SAA, and SAL have mean frequencies for the set of LFD alleles of 43%, 39%, and 49%, respectively (Table 7; the Aché population was excluded from these calculations due to its outlier status). For *DRB1* the results are even more extreme, with MEA, SAA, and SAL having mean values of LFD alleles over 55%. These results imply that, even for populations and loci in which endemic alleles are not common, the pool of alleles carried by Native American populations is frequently made up of alleles which are rare in other world populations, implying that Native Americans have a distinctive genetic profile at HLA loci. The exceptions to this pattern are the North American populations, which on average display the lowest frequencies of alleles in the LFD class among the Native Americans groups. The populations of North America show different demographic history than the other populations of America. Several studies indicate that they are derived from subsequent migration flow from Siberia up to 5,000 YBP [3, 5, 9]. This more intense migratory flow between North American and East Asian populations likely reflects the greater HLA allele sharing between these regions.

**Table 7:** Mean total frequency for endemic and LFD alleles.

Locus	All Regions	Geographic Region			
		NAM	MEA	SAA	SAL
	**mean frequency of Endemic alleles**				
HLA-A	0.016	0.000	0.018	0.050	0.006
HLA-B	0.083	0.000	0.068	0.067	0.128
HLA-C	0.006	0.009	0.009	0.006	0.004
HLA-DRB1	0.000	0.000	0.000	0.000	0.000
	**mean frequency of LFD alleles**				
HLA-A	0.403	0.268	0.508	0.237	0.451
HLA-B	0.402	0.106	0.430	0.390	0.487
HLA-C	0.078	0.089	0.124	0.000	0.079
HLA-DRB1	0.636	0.310	0.740	0.568	0.705

For each locus, the mean frequency for endemic and LFD alleles was computed over the set of populations in the geographic region. The first column refers to continental average.

The sum of frequencies for LFD and endemic alleles varies substantially among populations and loci. To quantitatively assess the contribution of demographic factors, we estimated the correlation between mean microsatellite heterozygosity and the total frequency of endemic and LFD alleles, across all populations (Table 2). With the exception of *HLA-C*, which has very few endemic or LFD alleles, all other loci show a strong negative correlation over all populations between mean microsatellite heterozygosity and endemic/LFD frequency. Squaring these correlations shows that microsatellite heterozygosity (or homozygosity, since the correlation is negative) accounts for a substantial fraction of the endemic and LFD variance (43%, 34%, 24% for *HLA-A*, *-B* and -*DRB1*). Taken together, these results show that demographic factors play a central role in explaining the abundance of LFD alleles in Native American populations.

#### Differentiation based on *F*_*ST*_

We quantified genetic differentiation among populations and regions by computing *F*_*ST*_ for both HLA and microsatellites. We first averaged all pairwise *F*_*ST*_ values within or between populations, to obtain estimates of mean pairwise *F*_*ST*_ for different geographic scales (Table 8). Nearly all pairs of regions have high levels of differentiation, particularly for contrasts involving SAL and NAM. The only case in which regions showed low differentiation was for the contrast between the single SIB population and the NAM populations. This result once again shows the pattern of secondary migratory flow between these regions.

**Table 8:** Fst among pairs of regions, averaged over all population pairs.

Comparison	Region(s)	HLA-A	HLA-B	HLA-C	HLA-DRB1	mean Msat	mean HLA
Within	MEA	0.135	0.095	0.118	0.071	0.047	0.105
**Region**	NAM	-0.003	0.023	0.007	0.024	0.030	0.013
	SAA	0.141	0.104	0.118	0.048	0.023	0.103
	SAL	0.083	0.115	0.129	0.189	0.077	0.129
Between	MEA:NAM	0.085	0.081	0.094	0.097	0.055	0.089
Region	MEA:SAA	0.162	0.121	0.111	0.082	0.038	0.119
	MEA:SAL	0.149	0.117	0.136	0.146	0.064	0.137
	MEA:SIB	0.077	0.109	0.112	0.138	0.074	0.109
	NAM:SAA	0.073	0.091	0.070	0.060	0.041	0.074
	NAM:SAL	0.129	0.118	0.080	0.130	0.070	0.114
	NAM:SIB	-0.011	0.050	0.030	0.078	0.041	0.037
	SAA:SAL	0.179	0.126	0.116	0.134	0.053	0.139
	SAA:SIB	0.067	0.085	0.092	0.112	0.058	0.089
	SAL:SIB	0.130	0.124	0.099	0.170	0.089	0.131

**Fig 6:**
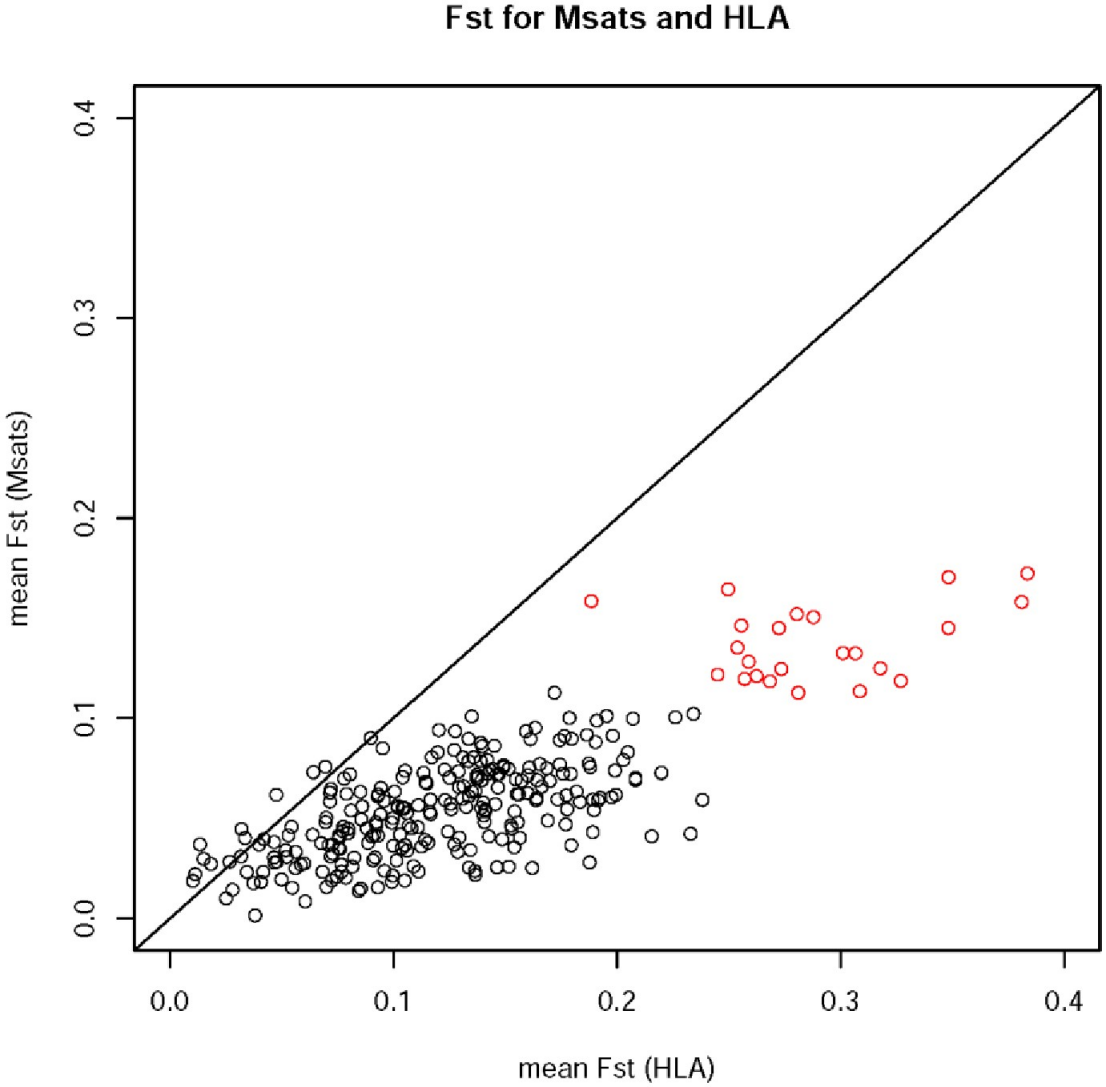
F_*ST*_ for population pairs at HLA and microsatellites. *F*_*ST*_ values were computed for each population pair and locus. *F*_*ST*_ values were summarized over the microsatellite and HLA loci separately. Results are shown for microsatellites in the highest (third) quartile of heterozygosity. Pairs involving the Ache are in red since this population has been excluded from other analyses due to their outlier status.

The *F*_*ST*_ values at HLA loci are consistently higher than those for microsatellites (Fig 6), a result which remains unchanged when the *R*_*ST*_ measure is used for the microsatellites, and despite the fact that our method controls for differences in heterozygosity values at these two sets of markers (see methods). The higher differentiation at HLA loci differs from the expectation under balancing selection, which is that of decreased differentiation relative to neutral markers [45]. These results suggest that HLA loci show high levels of variation, deviations from neutrality-equilibrium and relatively high levels of population differentiation, even though under balancing selection. This scenario is consistent with that described by Brandt et al. [47], in which populations of the same geographic region tend to show higher *F*_*ST*_ values for HLA SNPs, while populations of distinct geographic regions have lower *F*_*ST*_ values. Suggesting that local adaptation of HLA alleles may contribute to population differentiation on a regional scale, however, without erasing signatures of long-term balancing selection.

#### Standardized differentiation

**Fig 7:**
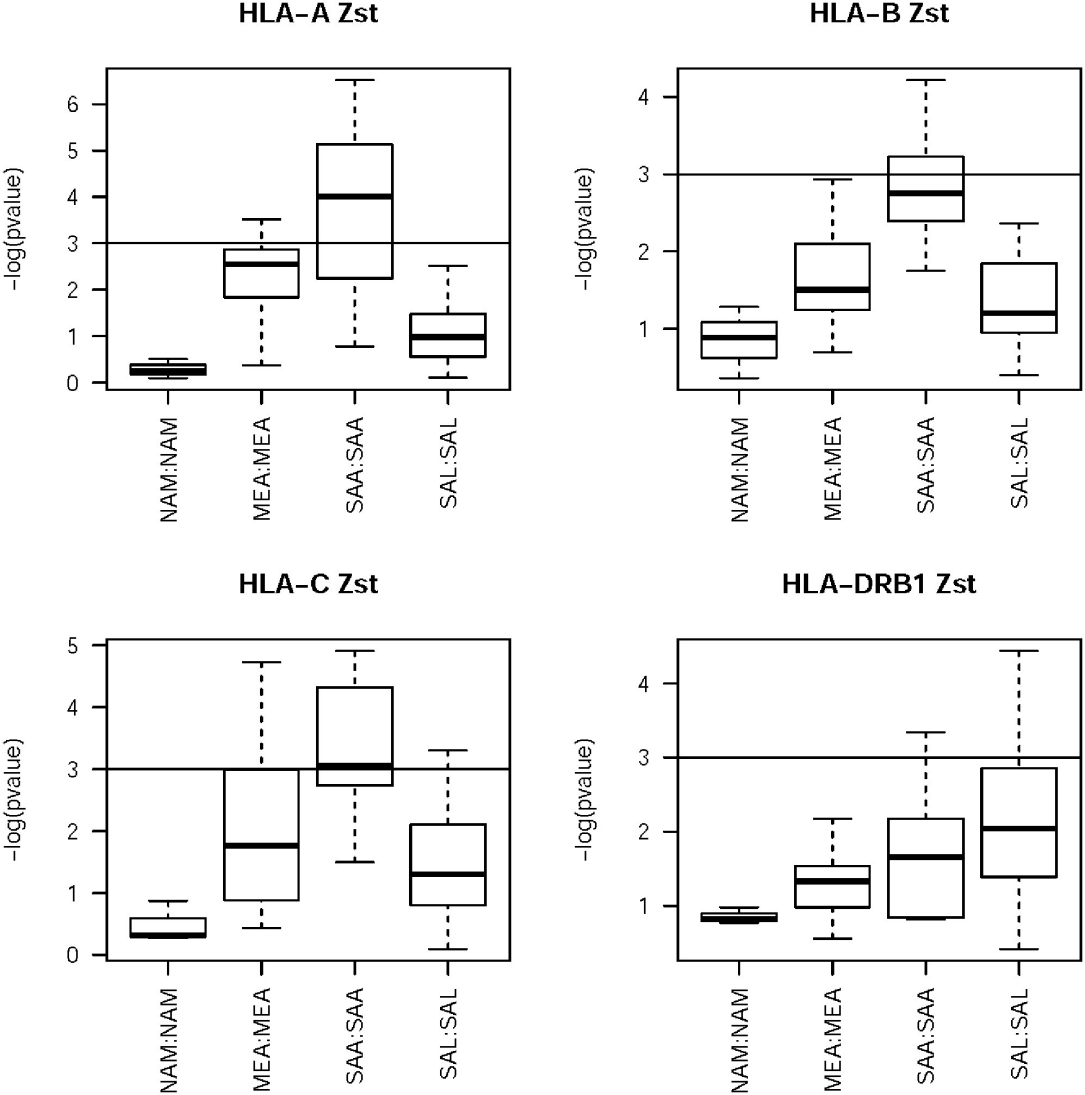
*Z*_*ST*_ values by region and locus. Empirical p-value from the distribution of microsatellite *F*_*ST*_*s*. *Z*_*ST*_ measures the number of standard deviations the HLA *F*_*ST*_ values are from that of the neutral loci. The line at–log(p-value) = 3 corresponds to the 0.05 level of significance.

Comparing the *F*_*ST*_ and standardized *Z*_*ST*_ values allows us to examine whether populations from different regions are more (or less) differentiated at HLA relative to the set of putatively neutral microsatellite loci (Fig 7). The general trend for HLA *F*_*ST*_ results is that the South American regions have larger *F*_*ST*_ values than the NAM and MEA populations, with the MEA populations slightly higher than NAM populations. Based on *Z*_*ST*_ results, the difference between MEA and NAM populations is slightly larger. In South America, the Andean populations stand out based on the *Z*_*ST*_ results, with much higher HLA differentiation relative to the microsatellite background.

Previous studies with uniparental and genome-wide data had also shown that Andean populations have higher within and between population variability than Amazonian natives [48–50]. Besides that, the Andeans show an excess of rare alleles with respect to the mutation-drift equilibrium expectation. This finding could be explained by the expansion of the Andean population after the rise of complex societies around 4,000 YBP and population displacements from conquests in the Inca Empire. Although Mesoamerica has gone through the same transition, followed by population growth, in this region the regional dynamics, with forced migration, led to greater genetic flow between Andean groups [50, 51]. This process has triggered low population differentiation for genomic markers, but not for HLA, suggesting that forces other than demography may be acting in this system.

### 3.6 Haplotype distributions in the Americas

In order to gain further insight into the history and dynamics of the endemic, large frequency difference, and non-LFD/non-endemic alleles, we analyzed the allele specific heterozygosity (ASH) for these allelic classes. ASH is the heterozygosity of alleles found in haplotypes with the allele that has been conditioned on. Because the allele specific heterozygosity is strongly affected by the heterozygosity of the conditioned upon locus (i.e., alleles at low frequencies typically have less diversity at linked sites) we stratified our analysis by frequency classes (Table 9).

**Table 9:** Allele specific heterozygosity by allele category.

Locus	Category of conditioned allele	Allele Specific Heterozygosity
A	LFD	0.254
B	LFD	0.120
C	LFD	0.201
DRB1	LFD	0.331
All loci	LFD (.05<freq <.10)	0.189
All loci	LFD (.10<freq)	0.393
A	Non-Endemic/LFD	0.293
B	Non-Endemic/LFD	0.156
C	Non-Endemic/LFD	0.222
DRB1	Non-Endemic/LFD	0.157
All loci	Non-Endemic/LFD (.05<freq <.10)	0.260
All loci	Non-Endemic/LFD (.10<freq)	0.444

Allele Specific Heterozygosity is the heterozygosity of alleles found in haplotypes with the allele that has been conditioned on. Values were averaged over all alleles of that type for each pair of loci.

Overall, we find that LFD alleles reside on less diverse haplotypes than non-LFD/non-endemic alleles, again regardless of the frequency of the conditioned upon allele (Table 9). This result is consistent with the endemic, LFD and non-LFD/non-endemic status of alleles serving as a proxy for allele age: younger alleles are expected to be more regionally restricted, and to be associated to a reduced number of haplotypes. Older alleles, on the other hand, are expected to be associated to diverse haplotypic contexts, as is the case for the non-LFD/non-endemic alleles. Also, in the presence of selection, alleles on a selected haplotype might be less diverse.

## 4. Conclusion

We have analyzed HLA variation in Native American populations while controlling for demographic history by using a large set of microsatellite loci from the same individuals. Our neutrality tests show that, as expected, there is evidence for long-term balancing selection at the HLA loci (as documented by Tajima’s test of neutrality). Thus, much of the signature of selection seen in America for HLA genes may reflect selection that took place much earlier, before America was occupied.

We also documented a higher degree of among-population genetic differentiation for HLA loci, as compared to microsatellites. Although this direct comparison is challenging to interpret due to the differences in mutational processes underlying these types of markers, we controlled for a major confounding factor upon differentiation, which is within population variability, and showed that the finding of increased differentiation was robust. Lindo et al. [52] also identified marked differentiation at an HLA locus (*DQA1*), but in the context of differences between an ancestral and a descendant population (based on sequencing of ancient samples). They argued that the pattern results from a shift of selective regimes, such that previously advantageous variants became negatively selected after European contact. Together, these results favor the interpretation that selection on HLA genes in the Americas involves a mixture of long-term balancing selection and a combination of episodes of more recent positive and balancing selection.

Andean populations stood out based on an empirically standardized measure of differentiation with much higher HLA differentiation relative to the microsatellite background for Class I loci. For genomic markers demographic events, including Incan Empire conquests and forced migrations, have been hypothesized for lower population differentiation observed among Andean populations. The higher differentiation for HLA class I loci suggests non-demographic forces have shaped these allele frequency distributions.

Our analyses are based on sequence level variation in exons 2 and 3 of class I loci, and exon 2 of the *HLA-DRB1* class II locus (see Methods). It is clear that contemporary techniques based on NGS can provide a higher resolution of HLA diversity, uncovering alleles defined by variation in exons, introns and regulatory sequences which were not surveyed in this study. Our analysis is not a complete molecular survey of HLA diversity in Native American populations, but it does provide a reliable survey of how HLA variation in exons 2 and 3 is related to neutral variation (captured by microsatellite polymorphism in the same samples). Our focus on a subset of coding regions also implies that alleles which we consider the same in America and in other regions of the world, may in fact have originated from different ancestral sequences. To distinguish such identify by state from common origin, it will be necessary to either survey a longer portion of each locus, or their haplotypic contexts.

Despite the evidence for selection seen in our dataset, we found that many features of HLA variation are accounted for by the recent demographic history of these populations. This is a recurrent feature of human evolutionary history, and there are in fact few cases where selection overrides the patterns of genetic differentiation which originate due to demographic processes [23]. Specifically, we show that the abundance of alleles which are unique to America (or much more common in this continent than others) is to a large degree explained by demographic history, as are overall levels of polymorphism. Thus, despite the intense selection pressures acting on HLA loci, recent demographic history has played a substantial role in shaping their overall patterns of variations within America.

## Supporting information

S1 Table (PDF)

S2 Table (PDF)
