# Supplementary material for: Demographic history and selection at HLA loci in Native Americans
Source: PLoS One. 2020 Nov 4;15(11):e0241282. doi: 10.1371/journal.pone.0241282 (PMC7641399; doi:10.1371/journal.pone.0241282)
Supplement: S1 Table — (PDF) [file pone.0241282.s001.pdf]

S1 Table: HLA genotypes

The pages in the table that follows contain the HLA genotypes for the 24 populations analyzed in Single RM, Meyer D, et al. "Demographic history and selection at HLA loci in Native Americans"

The accession number hyperlinks below link to this HLA data in the Allele Frequency Net Database (AFND). AFND is a repository for the storage of genetic data related to human leukocyte antigens (HLA), killer-cell immunoglobulin-like receptors (KIR), major histocompatibility complex Class I chain related genes (MIC) and a number of cytokine gene polymorphisms in worldwide populations. It is mantained by the Royal Liverpool and Broadgreen University Hospitals and the University of Liverpool.

http://www.allelefrequencies.net/population/AFND3692 TundraNentsi (SIB)
http://www.allelefrequencies.net/population/AFND3693 Wayuu (SAL)
http://www.allelefrequencies.net/population/AFND3694 Guarani (SAL)
http://www.allelefrequencies.net/population/AFND3695 Huilliche (SAA)
http://www.allelefrequencies.net/population/AFND3696 Inga (SAA)
http://www.allelefrequencies.net/population/AFND3697 Zapotec (MEA)
http://www.allelefrequencies.net/population/AFND3698 Ache (SAL)
http://www.allelefrequencies.net/population/AFND3699 TicunaTarapaca (SAL)
http://www.allelefrequencies.net/population/AFND3700 TicunaArara (SAL)
http://www.allelefrequencies.net/population/AFND3701 Waunana (SAL)
http://www.allelefrequencies.net/population/AFND3702 Embera (SAL)
http://www.allelefrequencies.net/population/AFND3703 Zenu (SAL)
http://www.allelefrequencies.net/population/AFND3704 Kogi (SAA)
http://www.allelefrequencies.net/population/AFND3705 Arhuaco (SAA)
http://www.allelefrequencies.net/population/AFND3706 Aymara (SAA)
http://www.allelefrequencies.net/population/AFND3707 Quechua (SAA)
http://www.allelefrequencies.net/population/AFND3708 Guaymi (MEA)
http://www.allelefrequencies.net/population/AFND3709 Cabecar (MEA)
http://www.allelefrequencies.net/population/AFND3710 Kaqchikel (MEA)
http://www.allelefrequencies.net/population/AFND3711 Mixe (MEA)
http://www.allelefrequencies.net/population/AFND3712 Mixtec (MEA)
http://www.allelefrequencies.net/population/AFND3713 Ojibwa (NAM)
http://www.allelefrequencies.net/population/AFND3714 Cree (NAM)
http://www.allelefrequencies.net/population/AFND3715 Chipewyan (NAM)

SIB=Siberia, NAM=N.America, MEA=Meso America, SAA=S.America Andes, SAL=S.America Lowlands

Note: DRB1*04:05:01/04:05:03 and DRB1*11:01:01/11:01:06 are alleles that differ in the last three codons of exon 2 that were not distinguished. B*15:01:01:01G-N represents B*15:01:01G alleles, excluding the B*15:01:01:02N allele after inspection of the intron sequence.

pop	id	A_1	A_2	B_1	B_2	C_1	C_2	DRB1_1	DRB1_2
Ache	2734	68:01:02G	68:01:02G	40:04	40:04	?	?	04:11	04:11
Ache	2735	02:11:01G	68:01:02G	40:04	48:02	03:04:01G	04:01:01G	04:11	04:11
Ache	2736	24:02:01G	68:01:02G	40:02:01G	40:04	03:04:01G	03:04:01G	04:11	08:07
Ache	2737	02:11:01G	02:11:01G	?	?	?	?	04:11	04:11
Ache	2738	68:01:02G	68:01:02G	40:04	48:02	?	?	04:11	04:11
Ache	2739	68:01:02G	68:01:02G	40:04	40:04	03:04:01G	03:04:01G	04:11	04:11
Ache	2743	02:01:01G	68:01:02G	39:05	40:04	03:04:01G	07:02:01G	04:11	14:02
Ache	2744	24:02:01G	68:01:02G	40:02:01G	40:04	03:04:01G	03:04:01G	04:11	08:07
Ache	2745	68:01:02G	68:01:02G	40:04	48:02	03:04:01G	04:01:01G	04:11	04:11
Ache	2748	02:11:01G	68:01:02G	40:03	40:04	03:04:01G	03:04:01G	04:11	14:13
Ache	2750	02:11:01G	68:01:02G	40:04	48:02	03:04:01G	04:01:01G	04:11	04:11
Ache	2751	02:11:01G	68:01:02G	?	?	?	?	?	?
Ache	2753	02:11:01G	68:01:02G	40:04	40:04	?	?	04:11	04:11
Arhuaco	2157	25:01:01	68:01:02G	18:01:01G	35:43:01G	12:03:01G	15:02:01G	14:06	15:02:01
Arhuaco	2158	25:01:01	68:01:02G	18:01:01G	35:43:01G	12:03:01G	15:02:01G	08:02:01	15:01:01
Arhuaco	2159	24:02:01G	25:01:01	18:01:01G	40:02:01G	03:05	12:03:01G	04:03:01	15:01:01
Arhuaco	2160	02:01:01G	02:22:01G	35:43:01G	35:43:01G	01:02:01G	01:02:01G	04:07:01	08:02:01
Arhuaco	2419	24:02:01G	25:01:01	18:01:01G	35:43:01G	01:02:01G	12:03:01G	08:02:01	15:01:01
Arhuaco	2420	24:02:01G	29:01:01G	40:02:01G	51:08	03:05	16:02	04:03:01	13:03:01
Arhuaco	2458	24:02:01G	24:02:01G	35:43:01G	35:99	01:02:01G	03:05	08:02:01	08:02:01
Arhuaco	2459	24:02:01G	24:02:01G	35:43:01G	35:43:01G	01:02:01G	01:02:01G	08:02:01	08:02:01
Arhuaco	2460	25:01:01	68:01:02G	15:01:01G-N	18:01:01G	01:02:01G	12:03:01G	04:07:01	15:01:01
Arhuaco	2575	24:02:01G	24:02:01G	27:05:02G	35:99	02:02:02	03:05	04:05:01/04:05:03	08:02:01
Arhuaco	2576	24:02:01G	32:01:01G	35:43:01G	35:99	01:02:01G	03:05	08:02:01	08:02:01
Arhuaco	2577	02:22:01G	24:02:01G	35:43:01G	35:43:01G	01:02:01G	01:02:01G	04:07:01	08:02:01
Arhuaco	2578	29:01:01G	68:01:02G	35:43:01G	51:08	15:02:01G	16:02	13:03:01	14:02
Arhuaco	2579	02:22:01G	24:03:01G	35:12	35:43:01G	01:02:01G	15:02:01G	04:07:01	08:02:01
Arhuaco	2580	24:02:01G	24:02:01G	35:43:01G	35:99	01:02:01G	03:05	04:07:01	08:02:01
Arhuaco	2732	02:22:01G	24:02:01G	35:43:01G	35:99	01:02:01G	03:05	04:07:01	08:02:01
Arhuaco	2733	24:02:01G	25:01:01	18:01:01G	35:99	03:05	12:03:01G	08:02:01	15:01:01
Aymara	2101	02:01:01G	24:02:01G	35:05	40:08	03:04:01G	04:01:01G	04:04	08:02:01
Aymara	2102	02:01:01G	24:02:01G	14:02:01	35:05	04:01:01G	08:02	01:01:01	08:02:01
Aymara	2103	02:01:01G	02:22:01G	35:05	40:02:01G	03:57	04:01:01G	08:02:01	08:02:01
Aymara	2104	02:01:01G	02:01:01G	35:01:01G	35:05	04:01:01G	04:01:01G	08:02:01	08:02:01
Aymara	2105	68:01:02G	68:17	35:05	35:21	04:01:01G	04:01:01G	04:04	16:02:01
Aymara	2106	02:01:01G	02:01:01G	35:04:01	48:01:01G	03:04:01G	04:01:01G	08:02:01	09:01:02
Aymara	2107	02:01:01G	68:17	35:19	40:08	03:04:01G	08:01:01	08:02:01	08:02:01
Aymara	2108	02:01:01G	24:02:01G	41:01	48:01:01G	03:04:01G	17:01:01G	09:01:02	13:02:01
Aymara	2109	02:01:01G	02:01:01G	41:01	51:01:01G	07:01:01G	15:02:01G	09:01:02	13:05:01
Aymara	2110	01:01:01G	02:01:01G	35:04:01	35:05	04:01:01G	04:01:01G	04:04	08:02:01
Aymara	2111	02:01:01G	02:22:01G	15:39	48:01:01G	01:02:01G	08:03	04:03:01	09:01:02
Aymara	2112	24:02:01G	68:01:02G	35:05	35:05	04:01:01G	04:01:01G	04:04	04:04
Aymara	2113	02:01:01G	02:01:01G	35:05	48:01:01G	04:01:01G	08:03	04:03:01	08:02:01
Aymara	2114	02:01:01G	02:01:01G	35:05	40:02:01G	03:04:01G	04:01:01G	08:02:01	14:02
Aymara	2115	02:01:01G	26:01:01G	35:01:01G	55:01:01G	03:03:01G	04:01:01G	03:01:01	04:07:01
Aymara	2116	02:01:01G	24:02:01G	35:05	35:05	04:01:01G	04:01:01G	04:04	14:02
Aymara	2117	02:01:01G	02:01:01G	48:01:01G	48:01:01G	15:02:01G	15:02:01G	09:01:02	09:01:02
Aymara	2118	24:02:01G	68:17	35:05	35:21	04:01:01G	04:01:01G	08:02:01	14:02
Aymara	2119	02:01:01G	68:17	35:05	35:19	04:01:01G	08:01:01	04:04	08:02:01
Aymara	2120	02:01:01G	24:02:01G	35:05	51:13:01	03:04:01G	04:01:01G	08:02:01	08:02:01
Cabecar	2022	02:06:01G	24:03:02	35:43:01G	40:02:01G	01:02:01G	03:05	04:07:01	16:02:01
Cabecar	2023	24:03:02	24:03:02	15:01:01G-N	35:43:01G	01:02:01G	01:02:01G	04:07:01	08:02:01
Cabecar	2024	02:06:01G	24:03:02	35:43:01G	40:02:01G	01:02:01G	03:05	16:02:01	04:07:01
Cabecar	2025	24:02:01G	68:01:02G	35:01:01G	40:02:01G	03:05	04:01:01G	04:03:01	04:07:01
Cabecar	2026	24:03:02	68:03:01	15:01:01G-N	40:02:01G	01:02:01G	03:05	04:07:01	08:02:01
Cabecar	2027	24:02:01G	68:01:02G	40:02:01G	40:02:01G	03:05	03:05	16:02:01	04:03:01
Cabecar	2028	24:02:01G	24:02:01G	40:02:01G	41:01	03:05	17:01:01G	13:02:01	04:07:01
Cabecar	2029	24:02:01G	24:02:01G	35:01:01G	40:02:01G	03:04:01G	03:05	16:02:01	14:02
Cabecar	2030	24:02:01G	68:30	40:02:01G	40:02:01G	03:05	03:05	16:02:01	04:03:01
Cabecar	2031	24:02:01G	24:03:02	35:01:01G	35:43:01G	01:02:01G	03:04:01G	14:02	04:07:01
Cabecar	2032	02:06:01G	24:02:01G	35:01:01G	40:02:01G	03:04:01G	03:05	16:02:01	14:02
Cabecar	2033	24:02:01G	24:03:02	35:01:01G	40:02:01G	03:04:01G	03:05	14:02	04:07:01
Cabecar	2034	24:02:01G	24:02:01G	15:01:01G-N	35:43:01G	01:02:01G	01:02:01G	16:02:01	04:07:01
Cabecar	2035	24:03:02	24:03:02	35:43:01G	35:43:01G	01:02:01G	01:02:01G	04:07:01	04:07:01
Cabecar	2036	23:01:01G	68:01:02G	40:02:01G	57:02	03:05	07:01:01G	04:07:01	07:01:01

Cabecar	2037	24:03:02	68:01:02G	35:43:01G	40:02:01G	01:02:01G	03:05	04:07:01	04:07:01
Cabecar	2038	68:01:02G	68:30	40:02:01G	40:02:01G	03:05	03:05	04:07:01	04:07:01
Cabecar	2039	02:06:01G	24:02:01G	15:01:01G-N	40:02:01G	01:02:01G	03:05	16:02:01	16:02:01
Cabecar	2040	68:01:02G	68:30	40:02:01G	40:02:01G	03:05	03:05	04:07:01	16:02:01
Chipewyan	2006	02:01:01G	24:02:01G	07:02:01G	49:01	03:04:01G	07:01:01G	07:01:01	11:01:01/11:01:06
Chipewyan	2012	02:01:01G	24:02:01G	35:01:01G	51:01:01G	04:01:01G	15:02:01G	14:01:01G	14:02
Chipewyan	2381	02:01:01G	24:02:01G	27:05:02G	35:01:01G	02:02:02	04:04:01	08:02:01	08:11
Chipewyan	2382	02:01:01G	24:02:01G	40:02:01G	44:02:01G	03:04:01G	05:01:01G	01:01:01	09:01:02
Chipewyan	2383	02:01:01G	02:06:01G	15:01:01G-N	39:01:01G	01:02:01G	07:02:01G	04:03:01	08:11
Chipewyan	2384	02:01:01G	02:01:01G	39:01:01G	44:02:01G	05:01:01G	12:03:01G	01:01:01	16:01:01
Chipewyan	2385	02:01:01G	26:01:01G	27:05:02G	38:01:01	02:02:02	12:03:01G	03:01:01	09:01:02
Chipewyan	2386	02:01:01G	02:01:01G	27:05:02G	35:01:01G	02:02:02	04:01:01G	09:01:02	14:01:01G
Chipewyan	2387	02:01:01G	24:02:01G	35:01:01G	40:02:01G	03:04:01G	04:01:01G	09:01:02	14:02
Chipewyan	2388	24:02:01G	24:02:01G	51:01:01G	51:01:01G	15:02:01G	15:02:01G	14:01:01G	14:01:01G
Chipewyan	2389	02:06:01G	02:06:01G	27:05:02G	40:02:01G	02:02:02	03:04:01G	09:01:02	14:02
Chipewyan	2390	02:01:01G	02:06:01G	15:01:01G-N	44:02:01G	01:02:01G	05:01:01G	01:01:01	09:01:02
Chipewyan	2391	02:01:01G	02:01:01G	39:01:01G	44:02:01G	05:01:01G	12:03:01G	13:01:01	16:01:01
Chipewyan	2392	24:02:01G	31:01:02G	35:01:01G	51:01:01G	04:01:01G	14:02:01	09:01:02	14:02
Chipewyan	2393	02:01:01G	02:06:01G	39:01:01G	40:02:01G	03:04:01G	12:03:01G	14:02	16:01:01
Chipewyan	2394	02:01:01G	24:02:01G	07:02:01G	40:02:01G	03:04:01G	07:02:01G	09:01:02	15:01:01
Chipewyan	2395	02:01:01G	24:02:01G	15:01:01G-N	44:02:01G	01:02:01G	05:01:01G	01:01:01	14:02
Chipewyan	2396	02:01:01G	02:01:01G	27:05:02G	44:02:01G	02:02:02	05:01:01G	01:01:01	08:02:01
Chipewyan	2397	01:01:01G	03:01:01G	27:05:02G	37:01:01	02:02:02	06:02:01G	11:04:01	15:01:01
Chipewyan	2398	02:01:01G	02:01:01G	27:05:02G	35:01:01G	02:02:02	04:01:01G	09:01:02	14:01:01G
Chipewyan	2399	24:02:01G	31:01:02G	35:01:01G	35:01:01G	04:01:01G	04:04:01	04:07:01	14:02
Chipewyan	2400	24:02:01G	31:01:02G	40:02:01G	51:01:01G	03:04:01G	14:02:01	09:01:02	14:01:01G
Chipewyan	2455	02:01:01G	24:02:01G	40:02:01G	49:01	03:04:01G	07:01:01G	11:01:01/11:01:06	14:02
Chipewyan	2520	02:01:01G	03:01:01G	07:02:01G	07:02:01G	07:02:01G	07:02:01G	13:01:01	15:01:01
Chipewyan	2800	01:01:01G	02:01:01G	08:01:01G	39:01:01G	07:01:01G	12:03:01G	03:01:01	16:01:01
Cree	2401	02:01:01G	24:02:01G	39:06:02	51:01:01G	07:02:01G	07:02:01G	04:10	08:11
Cree	2402	02:01:01G	03:01:01G	51:01:01G	51:01:01G	07:02:01G	15:02:01G	04:01:01	04:07:01
Cree	2403	02:01:01G	02:06:01G	27:05:02G	35:01:01G	02:02:02	04:01:01G	14:02	16:02:01
Cree	2404	02:01:01G	24:02:01G	51:01:01G	51:01:01G	14:02:01	16:02	09:01:02	11:02:01
Cree	2405	02:06:01G	24:02:01G	07:05:01G	40:01:01G	03:04:01G	15:05:01G	11:03	14:02
Cree	2406	01:01:01G	02:01:01G	08:01:01G	51:01:01G	07:01:01G	15:02:01G	03:01:01	04:04
Cree	2407	23:01:01G	24:02:01G	49:01	51:01:01G	07:01:01G	14:02:01	09:01:02	11:04:01
Cree	2408	02:06:01G	02:06:01G	35:01:01G	51:01:01G	04:01:01G	04:01:01G	04:07:01	08:11
Cree	2409	01:01:01G	02:01:01G	27:05:02G	37:01:01	02:02:02	06:02:01G	07:01:01	14:02
Cree	2410	02:01:01G	31:01:02G	51:01:01G	57:01:01	06:16N	15:02:01G	04:01:01	04:07:01
Cree	2411	02:01:01G	02:06:01G	40:01:01G	57:01:01	03:04:01G	06:02:01G	04:07:01	07:01:01
Cree	2412	02:01:01G	02:06:01G	07:02:01G	35:01:01G	04:01:01G	07:02:01G	04:10	15:02:01
Cree	2413	24:02:01G	24:02:01G	35:01:01G	35:01:01G	04:01:01G	04:04:01	04:10	08:11
Cree	2414	24:02:01G	29:02:01	14:01	40:02:01G	03:04:01G	08:02	07:01:01	14:01:01G
Cree	2415	02:01:01G	31:01:02G	35:01:01G	51:01:01G	04:01:01G	15:02:01G	04:07:01	16:02:01
Cree	2416	24:02:01G	31:01:02G	48:01:01G	51:01:01G	03:04:01G	08:01:01	04:07:01	04:07:01
Cree	2417	31:01:02G	32:01:01G	27:05:02G	44:02:01G	02:02:02	05:01:01G	09:01:02	09:01:02
Cree	2418	02:01:01G	02:01:01G	07:02:01G	57:01:01	06:02:01G	07:02:01G	07:01:01	15:02:01
Embera	2561	24:02:01G	31:01:02G	39:05	40:02:01G	03:04:01G	07:02:01G	04:07:01	04:07:01
Embera	2562	24:02:01G	31:01:02G	35:10	40:02:01G	03:04:01G	03:04:01G	16:02:01	04:07:01
Embera	2563	24:02:01G	68:01:02G	39:05	39:11	07:02:01G	07:02:01G	14:02	04:07:01
Embera	2564	02:13	24:02:01G	39:05	39:05	07:02:01G	07:02:01G	04:07:01	04:11
Embera	2565	02:13	24:02:01G	39:05	39:05	07:02:01G	07:02:01G	04:07:01	04:11
Embera	2566	02:13	24:02:01G	39:05	39:05	07:02:01G	07:02:01G	04:07:01	04:11
Embera	2567	24:02:01G	24:02:01G	35:10	39:05	03:04:01G	07:02:01G	16:02:01	04:07:01
Embera	2568	24:02:01G	68:01:02G	35:10	39:11	03:04:01G	07:02:01G	16:02:01	16:02:01
Embera	2569	24:02:01G	24:03:01G	35:04:01	40:02:01G	03:04:01G	04:01:01G	14:02	04:04
Embera	2570	24:02:01G	24:03:01G	35:04:01	35:10	03:04:01G	04:01:01G	16:02:01	14:02
Embera	2571	11:01:01G	24:02:01G	35:12	51:01:01G	04:01:01G	15:02:01G	07:01:01	16:02:01
Embera	2572	02:11:01G	24:02:01G	39:05	40:04	03:04:01G	07:02:01G	14:02	04:07:01
Embera	2573	24:03:01G	24:03:01G	39:05	40:04	03:04:01G	15:10:01	14:02	04:07:01
Embera	2574	24:02:01G	24:02:01G	35:10	39:05	03:04:01G	07:02:01G	16:02:01	04:07:01
Guarani	2720	02:01:01G	02:11:01G	15:01:01G-N	15:04	?	?	?	?
Guarani	2721	02:11:01G	24:02:01G	39:05	40:04	03:04:01G	07:02:01G	14:02	04:07:01
Guarani	2722	02:01:01G	24:03:01G	15:01:01G-N	40:04	03:03:01G	03:04:01G	11:04:01	08:04:01
Guarani	2723	68:02:01G	68:02:01G	14:02:01	15:04	08:02	15:02:01G	13:03:01	14:02
Guarani	2724	02:01:01G	68:01:02G	15:04	15:04	03:03:01G	03:03:01G	16:02:01	16:02:01

Guarani	2725	02:01:01G	24:03:01G	40:04	40:04	03:04:01G	15:02:01G	11:04:01	14:02
Guarani	2726	02:01:01G	02:12	15:04	35:04:01	03:03:01G	03:04:01G	16:02:01	14:02
Guarani	2727	24:02:01G	24:03:01G	15:04	40:02:01G	03:03:01G	03:04:01G	16:02:01	16:02:01
Guarani	2728	02:01:01G	24:02:01G	40:04	52:01:02	04:01:01G	15:02:01G	14:02	04:11
Guarani	2729	02:01:01G	31:15	15:04	52:01:01G	15:02:01G	15:02:01G	16:02:01	14:02
Guaymi	2001	02:22:01G	24:02:01G	35:01:01G	35:43:01G	01:02:01G	04:01:01G	04:05:04	04:07:01
Guaymi	2002	02:22:01G	68:01:02G	35:01:01G	40:02:01G	03:05	04:01:01G	04:05:04	08:02:01
Guaymi	2003	02:22:01G	24:02:01G	35:01:01G	40:02:01G	03:05	04:01:01G	04:05:04	04:07:01
Guaymi	2004	02:22:01G	24:02:01G	35:01:01G	51:01:01G	04:01:01G	04:01:01G	16:02:01	04:05:04
Guaymi	2005	24:02:01G	68:01:02G	35:43:01G	40:02:01G	01:02:01G	03:05	04:07:01	08:02:01
Guaymi	2007	02:22:01G	02:01:01G	35:01:01G	35:49	04:01:01G	04:01:01G	04:05:04	04:05:04
Guaymi	2008	02:22:01G	31:01:02G	15:01:01G-N	35:01:01G	01:02:01G	04:01:01G	16:02:01	04:05:04
Guaymi	2009	24:02:01G	68:01:02G	35:43:01G	40:02:01G	01:02:01G	03:05	04:07:01	08:02:01
Guaymi	2010	02:22:01G	02:22:01G	35:01:01G	35:01:01G	04:01:01G	04:01:01G	04:05:04	04:05:04
Guaymi	2011	24:02:01G	31:01:02G	15:01:01G-N	35:102	01:02:01G	01:02:01G	16:02:01	04:07:01
Guaymi	2013	02:22:01G	24:02:01G	35:01:01G	40:02:01G	03:05	04:01:01G	04:05:04	04:07:01
Guaymi	2014	02:22:01G	24:02:01G	35:01:01G	35:43:01G	01:02:01G	04:01:01G	04:05:04	04:07:01
Guaymi	2015	02:22:01G	24:02:01G	35:01:01G	35:43:01G	01:02:01G	04:01:01G	04:05:04	04:07:01
Guaymi	2016	02:22:01G	24:02:01G	35:01:01G	35:43:01G	01:02:01G	04:01:01G	04:05:04	14:02
Guaymi	2017	02:22:01G	24:02:01G	35:43:01G	40:02:01G	01:02:01G	03:05	16:02:01	04:07:01
Guaymi	2018	02:22:01G	24:02:01G	35:01:01G	35:43:01G	01:02:01G	04:01:01G	04:05:04	04:07:01
Guaymi	2019	02:22:01G	24:02:01G	35:01:01G	35:43:01G	01:02:01G	04:01:01G	04:05:04	04:07:01
Guaymi	2020	02:22:01G	30:01:01G	35:01:01G	35:43:01G	01:02:01G	04:01:01G	04:05:04	04:07:01
Huilliche	2121	24:02:01G	24:02:01G	39:09	39:09	07:02:01G	07:02:01G	14:02	14:02
Huilliche	2122	02:01:01G	68:01:02G	39:09	39:09	?	?	04:07:01	16:02:01
Huilliche	2123	02:01:01G	68:16	39:09	39:09	?	?	08:02:01	08:02:01
Huilliche	2124	01:01:01G	68:23	08:01:01G	40:02:01G	03:04:01G	07:01:01G	08:02:01	11:01:01/11:01:06
Huilliche	2125	02:01:01G	24:02:01G	39:09	39:09	07:02:01G	07:02:01G	08:02:01	16:02:01
Huilliche	2126	68:01:02G	68:01:02G	44:02:01G	51:01:01G	03:04:01G	05:01:01G	08:01:01G	09:01:02
Huilliche	2127	01:01:01G	31:01:02G	35:09:01	57:01:01	04:01:01G	06:02:01G	04:02	04:07:01
Huilliche	2128	11:01:01G	24:02:01G	14:01	35:01:01G	07:02:01G	08:02	07:01:01	14:01:01G
Huilliche	2129	24:02:01G	29:02:01	39:09	44:03:01G	?	?	03:01:01	04:07:01
Huilliche	2130	02:01:01G	68:01:02G	15:01:01G-N	35:09:01	04:01:01G	07:02:01G	04:07:01	04:07:01
Huilliche	2131	02:01:01G	68:01:02G	39:09	39:09	?	?	04:07:01	08:02:01
Huilliche	2132	68:01:02G	68:47	51:01:01G	51:01:01G	15:02:01G	15:02:01G	04:07:01	09:01:02
Huilliche	2133	02:01:01G	68:01:02G	39:01:01G	39:09	07:02:01G	07:02:01G	08:02:01	08:02:01
Huilliche	2134	03:01:01G	68:01:02G	15:01:01G-N	49:01	01:02:01G	07:01:01G	04:07:01	11:02:01
Huilliche	2135	68:01:02G	68:01:02G	39:09	51:01:01G	07:02:01G	15:02:01G	04:07:01	16:02:01
Huilliche	2136	24:02:01G	29:02:01	39:09	58:01:01G	07:01:01G	07:02:01G	08:04:01	14:02
Huilliche	2137	68:01:02G	68:01:02G	39:09	51:01:01G	07:02:01G	15:02:01G	04:07:01	14:02
Huilliche	2138	02:01:01G	68:01:02G	15:01:01G-N	39:09	?	?	14:02	16:02:01
Huilliche	2139	02:01:01G	03:01:01G	39:09	44:03:01G	07:02:01G	16:01:01	01:02:01	16:02:01
Huilliche	2140	02:01:01G	31:01:02G	35:09:01	51:01:01G	04:01:01G	15:02:01G	04:07:01	14:02
Inga	2501	02:13	11:01:01G	14:01	51:01:01G	08:02	15:02:01G	04:03:01	13:01:01
Inga	2502	24:02:01G	24:02:01G	?	?	01:02:01G	15:02:01G	?	?
Inga	2503	24:02:01G	24:02:01G	15:01:01G-N	15:08	01:02:01G	15:02:01G	04:04	04:07:01
Inga	2504	02:11:01G	31:01:02G	40:02:01G	48:01:01G	03:04:01G	08:03	04:04	04:11
Inga	2505	24:02:01G	26:01:01G	38:01:01	39:05	07:02:01G	12:03:01G	13:01:01	16:02:01
Inga	2506	02:11:01G	02:13	15:01:01G-N	40:02:01G	01:02:01G	03:04:01G	04:04	08:02:01
Inga	2507	24:02:01G	24:02:01G	35:43:01G	40:02:01G	01:02:01G	01:02:01G	04:07:01	04:07:01
Inga	2508	02:11:01G	11:01:01G	14:01	15:01:01G-N	01:02:01G	08:02	04:04	13:01:01
Inga	2509	02:11:01G	02:11:01G	40:04	48:01:01G	03:04:01G	08:01:01	09:01:02	14:02
Inga	2510	02:11:01G	02:13	15:01:01G-N	40:02:01G	01:02:01G	03:04:01G	04:04	08:02:01
Inga	2512	02:01:01G	02:13	40:02:01G	40:02:01G	03:04:01G	03:04:01G	08:02:01	09:01:02
Inga	2513	24:02:01G	29:02:01	15:07	50:01	01:02:01G	06:02:01G	01:01:01	08:02:01
Inga	2514	02:01:01G	24:02:01G	40:02:01G	40:02:01G	03:04:01G	03:04:01G	08:02:01	08:02:01
Inga	2516	02:11:01G	24:02:01G	15:01:01G-N	35:43:01G	01:02:01G	01:02:01G	04:04	04:07:01
Inga	2517	01:01:01G	24:02:01G	35:43:01G	58:01:01G	01:02:01G	07:01:01G	04:07:01	13:02:01
Inga	2518	?	?	?	?	?	?	04:04	13:02:01
Kaqchikel	2681	24:02:01G	68:03:01	35:01:01G	39:08	07:02:01G	07:02:01G	04:08	16:02:01
Kaqchikel	2682	02:01:01G	26:01:01G	38:01:01	51:01:01G	12:03:01G	14:02:01	11:01:01/11:01:06	14:01:01G
Kaqchikel	2683	24:02:01G	68:03:01	35:01:01G	39:05	03:04:01G	07:02:01G	04:07:01	04:10
Kaqchikel	2684	02:01:01G	66:01	41:01	57:01:01	06:02:01G	17:01:01G	03:01:01	04:02
Kaqchikel	2685	24:02:01G	25:01:01	39:06:02	44:02:01G	05:01:01G	07:02:01G	11:01:01/11:01:06	14:06
Kaqchikel	2686	02:01:01G	68:03:01	39:02:02	39:05	07:02:01G	07:02:01G	04:07:01	16:02:01
Kaqchikel	2687	02:01:01G	24:02:01G	39:05	40:02:01G	03:04:01G	07:02:01G	08:02:01	14:06

Kaqchikel	2688	02:01:01G	24:02:01G	35:01:01G	35:12	03:04:01G	04:01:01G	04:07:01	04:07:01
Kaqchikel	2689	02:01:01G	24:02:01G	35:01:01G	39:02:02	04:01:01G	07:02:01G	04:07:01	14:02
Kaqchikel	2690	02:01:01G	30:02:01G	18:01:01G	35:12	04:01:01G	05:01:01G	03:01:01	08:02:01
Kaqchikel	2691	03:01:01G	24:02:01G	35:12	44:02:01G	04:01:01G	05:01:01G	04:05:01/04:05:03	08:02:01
Kaqchikel	2692	02:01:01G	30:01:01G	42:01	51:01:01G	15:02:01G	17:01:01G	08:02:01	13:02:01
Kaqchikel	2693	24:02:01G	68:03:01	35:01:01G	35:01:01G	03:04:01G	07:02:01G	04:07:01	14:02
Kaqchikel	2694	30:02:01G	68:01:02G	18:01:01G	35:17	04:01:01G	05:01:01G	03:01:01	14:06
Kaqchikel	2695	11:01:01G	26:01:01G	27:05:02G	38:01:01	02:07	12:03:01G	09:01:02	11:04:01
Kaqchikel	2696	24:02:01G	68:03:01	35:12	35:48	04:01:01G	07:02:01G	04:07:01	08:02:01
Kaqchikel	2697	02:01:01G	02:01:01G	35:01:01G	35:43:01G	01:02:01G	04:01:01G	04:07:01	14:06
Kaqchikel	2698	29:02:01	31:01:02G	40:02:01G	44:03:01G	03:05	16:01:01	13:01:01	04:07:01
Kaqchikel	2699	68:01:02G	68:05	35:43:01G	51:13:02	01:02:01G	08:01:01	04:04	04:07:01
Kogi	2461	24:02:01G	24:02:01G	15:01:01G-N	40:02:01G	01:02:01G	03:05	14:02	04:07:01
Kogi	2462	31:01:02G	68:01:02G	15:01:01G-N	35:01:01G	01:02:01G	04:01:01G	14:02	04:07:01
Kogi	2463	25:01:01	29:01:01G	18:01:01G	51:08	12:03:01G	16:02	15:01:01	13:03:01
Kogi	2464	24:02:01G	68:01:02G	15:01:01G-N	40:02:01G	01:02:01G	03:05	14:02	04:07:01
Kogi	2465	24:02:01G	31:01:02G	35:43:01G	40:02:01G	01:02:01G	03:05	04:03:01	04:07:01
Kogi	2466	24:02:01G	31:01:02G	35:43:01G	40:02:01G	01:02:01G	03:05	04:03:01	04:07:01
Kogi	2467	24:02:01G	31:01:02G	35:43:01G	40:02:01G	01:02:01G	03:05	04:03:01	04:07:01
Kogi	2468	24:02:01G	24:02:01G	35:43:01G	40:02:01G	01:02:01G	03:04:01G	04:07:01	08:02:01
Kogi	2469	24:02:01G	31:01:02G	35:43:01G	40:02:01G	01:02:01G	03:05	04:03:01	04:07:01
Kogi	2470	24:02:01G	24:02:01G	35:43:01G	40:02:01G	01:02:01G	01:02:01G	04:07:01	08:02:01
Kogi	2471	24:02:01G	31:01:02G	35:43:01G	35:43:01G	01:02:01G	01:02:01G	04:07:01	04:07:01
Kogi	2472	31:01:02G	31:01:02G	35:01:01G	35:43:01G	01:02:01G	04:01:01G	14:02	04:07:01
Kogi	2476	31:01:02G	68:01:02G	?	?	?	?	?	?
Kogi	2479	24:02:01G	31:01:02G	35:43:01G	40:02:01G	01:02:01G	01:02:01G	04:07:01	08:02:01
Kogi	2480	31:01:02G	68:01:02G	35:43:01G	35:43:01G	01:02:01G	15:02:01G	14:02	04:07:01
Mixe	2061	01:01:01G	02:01:01G	35:12	57:01:01	04:01:01G	07:01:01G	04:07:01	07:01:01
Mixe	2062	02:06:01G	02:06:01G	35:01:01G	40:02:01G	07:02:01G	15:02:01G	14:02	14:06
Mixe	2063	24:02:01G	24:02:01G	35:14:01	35:17	04:01:01G	04:01:01G	16:02:01	16:02:01
Mixe	2064	02:06:01G	02:06:01G	35:12	39:02:02	03:04:01G	04:01:01G	04:04	08:02:01
Mixe	2065	02:01:01G	31:01:02G	35:12	39:02:02	04:01:01G	07:02:01G	16:02:01	08:02:01
Mixe	2066	02:01:01G	02:01:01G	35:12	39:02:02	04:01:01G	07:02:01G	16:02:01	08:02:01
Mixe	2067	02:06:01G	31:01:02G	39:02:02	40:02:01G	03:04:01G	03:04:01G	04:07:01	08:02:01
Mixe	2068	02:06:01G	02:06:01G	35:12	52:01:02	03:03:01G	04:01:01G	16:02:01	08:02:01
Mixe	2069	02:01:01G	02:06:01G	35:17	39:02:02	03:04:01G	04:01:01G	14:06	04:04
Mixe	2070	02:01:01G	02:06:01G	35:17	40:02:01G	04:01:01G	15:02:01G	14:06	14:06
Mixe	2071	02:01:01G	02:06:01G	35:14:01	39:02:02	04:01:01G	07:02:01G	16:02:01	16:02:01
Mixe	2072	02:06:01G	31:01:02G	35:01:01G	39:02:02	04:01:01G	07:02:01G	16:02:01	04:04
Mixe	2073	02:06:01G	31:01:02G	40:02:01G	40:11	03:04:01G	15:02:01G	14:06	08:02:01
Mixe	2074	02:01:01G	31:01:02G	39:02:02	40:02:01G	03:04:01G	07:02:01G	16:02:01	16:02:01
Mixe	2075	02:01:01G	02:01:01G	35:01:01G	35:14:01	04:01:01G	04:01:01G	16:02:01	08:02:01
Mixe	2076	02:01:01G	02:06:01G	39:02:02	39:05	07:02:01G	07:02:01G	16:02:01	04:07:01
Mixe	2077	02:01:01G	31:01:02G	39:02:02	39:02:02	07:02:01G	07:02:01G	16:02:01	08:02:01
Mixe	2078	24:02:01G	31:01:02G	35:01:01G	40:02:01G	03:05	04:01:01G	14:02	04:07:01
Mixe	2079	02:01:01G	02:06:01G	35:12	39:02:02	03:04:01G	04:01:01G	04:04	08:02:01
Mixe	2080	02:06:01G	31:01:02G	39:02:02	39:02:02	03:04:01G	07:02:01G	16:02:01	04:04
Mixtec	2041	02:06:01G	24:02:01G	39:01:01G	39:05	03:04:01G	07:02:01G	14:06	04:04
Mixtec	2042	02:06:01G	02:06:01G	35:01:01G	39:05	07:02:01G	07:02:01G	04:07:01	08:02:01
Mixtec	2043	02:06:01G	24:02:01G	39:05	40:02:01G	03:05	07:02:01G	04:07:01	04:07:01
Mixtec	2044	02:06:01G	02:06:01G	35:01:01G	35:12	04:01:01G	04:01:01G	04:03:01	04:07:01
Mixtec	2045	31:01:02G	68:01:02G	35:01:01G	39:06:02	07:02:01G	07:02:01G	14:06	04:07:01
Mixtec	2046	02:01:01G	02:06:01G	35:12	39:01:01G	04:01:01G	07:02:01G	16:02:01	08:02:01
Mixtec	2047	24:02:01G	26:01:01G	35:14:01	38:01:01	04:01:01G	12:03:01G	16:01:01	16:02:01
Mixtec	2048	02:06:01G	02:06:01G	35:12	35:23	03:03:01G	04:01:01G	14:06	04:07:01
Mixtec	2049	02:06:01G	02:06:01G	35:12	39:05	04:01:01G	07:02:01G	04:07:01	04:07:01
Mixtec	2050	02:06:01G	02:06:01G	39:05	40:02:01G	03:06	07:02:01G	04:03:01	08:02:01
Mixtec	2051	02:06:01G	24:02:01G	35:23	39:06:02	03:03:01G	07:02:01G	14:06	14:06
Mixtec	2052	02:06:01G	68:01:02G	35:01:01G	35:23	03:03:01G	07:02:01G	14:06	04:07:01
Mixtec	2053	02:06:01G	31:01:02G	35:23	39:01:01G	03:03:01G	07:02:01G	16:02:01	14:06
Mixtec	2054	24:02:01G	68:01:02G	35:01:01G	35:12	04:01:01G	07:02:01G	04:07:01	08:02:01
Mixtec	2055	02:06:01G	24:02:01G	35:12	35:12	04:01:01G	04:01:01G	08:02:01	08:02:01
Mixtec	2056	24:02:01G	68:03:01	39:05	40:02:01G	03:04:01G	07:02:01G	16:02:01	04:07:01
Mixtec	2057	02:06:01G	24:02:01G	35:12	40:11	03:04:01G	04:01:01G	04:07:01	08:02:01
Mixtec	2058	02:06:01G	02:01:01G	35:12	39:02:02	04:01:01G	07:02:01G	16:02:01	04:07:01
Mixtec	2059	24:02:01G	31:01:02G	35:12	35:17	04:01:01G	04:01:01G	08:02:01	08:02:01

Mixtec	2060	02:01:01G	68:03:01	39:05	51:01:01G	07:02:01G	15:09	04:07:01	08:02:01
Ojibwa	2421	02:06:01G	24:02:01G	15:01:01G-N	40:02:01G	01:02:01G	03:04:01G	08:02:01	09:01:02
Ojibwa	2422	01:01:01G	02:06:01G	18:01:01G	40:01:01G	03:04:01G	05:01:01G	01:01:01	14:02
Ojibwa	2423	02:01:01G	02:01:01G	35:01:01G	44:08	04:04:01	05:01:01G	04:01:01	04:07:01
Ojibwa	2424	24:02:01G	31:01:02G	40:02:01G	51:01:01G	03:04:01G	15:02:01G	04:07:01	14:01:01G
Ojibwa	2425	24:02:01G	31:01:02G	39:01:01G	40:02:01G	03:04:01G	12:03:01G	01:01:01	09:01:02
Ojibwa	2426	24:02:01G	31:01:02G	35:01:01G	51:01:01G	04:01:01G	04:04:01	04:04	04:07:01
Ojibwa	2427	02:01:01G	26:01:01G	35:01:01G	40:01:01G	03:04:01G	04:01:01G	01:01:01	08:11
Ojibwa	2428	02:01:01G	02:06:01G	27:05:02G	35:01:01G	02:02:02	04:04:01	04:07:01	14:02
Ojibwa	2429	31:01:02G	68:01:02G	40:01:01G	48:07	03:04:01G	08:03	04:04	14:02
Ojibwa	2430	01:01:01G	02:01:01G	08:01:01G	35:01:01G	04:04:01	07:01:01G	03:01:01	08:11
Ojibwa	2431	01:01:01G	02:01:01G	35:01:01G	44:03:01G	04:04:01	16:01:01	07:01:01	08:11
Ojibwa	2432	31:01:02G	68:01:02G	42:02	48:07	08:03	17:01:01G	08:04:01	14:02
Ojibwa	2433	02:01:01G	03:01:01G	35:01:01G	40:02:01G	03:04:01G	04:01:01G	01:01:01	08:11
Ojibwa	2434	02:01:01G	02:01:01G	39:01:01G	39:01:01G	07:02:01G	07:02:01G	04:07:01	08:11
Ojibwa	2439	02:06:01G	24:02:01G	27:05:02G	35:01:01G	02:02:02	04:04:01	08:11	14:02
Ojibwa	2440	?	?	?	?	?	?	08:11	14:02
Quechua	x1	02:01:01G	33:01	14:02:01	48:01:01G	08:01:01	08:02	01:02	14:02
Quechua	x10	02:07:01G	24:02:01G	35:01:01G	46:01:01	01:02:01G	04:01:01G	04:07	09:01
Quechua	x11	02:01:01G	24:02:01G	35:05	40:02:01G	02:02:02	04:01:01G	11:01	14:02
Quechua	x12	02:01:01G	02:01:01G	15:04	39:05	01:02:01G	07:02:01G	04:04	14:06
Quechua	x13	02:01:01G	23:01:01G	35:09:01	50:01	04:01:01G	06:02:01G	03:01	09:01
Quechua	x14	02:01:01G	24:02:01G	35:01:01G	35:09:01	04:01:01G	04:01:01G	04:04	09:01
Quechua	x15	02:01:01G	02:01:01G	15:04	15:04	01:02:01G	01:02:01G	14:02	16:02
Quechua	x16	02:01:01G	02:01:01G	48:01:01G	51:01:01G	08:01:01	15:02:01G	09:01	09:01
Quechua	x17	02:01:01G	24:02:01G	15:01:01G-N	35:10	01:02:01G	04:01:01G	08:02	09:01
Quechua	x18	02:01:01G	02:01:01G	15:04	40:04	01:02:01G	03:04:01G	04:07	09:01
Quechua	x19	02:11:01G	24:02:01G	35:05	40:02:01G	03:04:01G	04:01:01G	04:07	08:02
Quechua	x2	02:01:01G	02:13	35:05	48:01:01G	03:04:01G	08:01:01	14:02	16:02
Quechua	x20	02:01:01G	02:01:01G	15:01:01G-N	40:04	01:02:01G	03:04:01G	08:02	09:01
Quechua	x21	02:01:01G	02:01:01G	15:04	35:09:01	01:02:01G	04:01:01G	09:01	09:01
Quechua	x3	02:22:01G	24:02:01G	35:05	44:03:01G	04:01:01G	04:01:01G	04:07	15:03
Quechua	x4	02:01:01G	02:64	07:02:01G	51:01:01G	07:02:01G	15:02:01G	07:01	09:01
Quechua	x5	02:01:01G	29:02:01	14:02:01	15:04	08:02	15:02:01G	04:07	11:01
Quechua	x6	02:01:01G	02:01:01G	15:05	40:64	01:02:01G	03:04:01G	08:02	09:01
Quechua	x7	02:01:01G	02:01:01G	15:04	15:05	01:02:01G	01:02:01G	09:01	09:01
Quechua	x8	02:01:01G	02:01:01G	35:05	35:05	04:01:01G	04:01:01G	04:04	14:02
Quechua	x9	02:01:01G	02:01:01G	39:09	48:01:01G	07:02:01G	08:03	08:02	14:06
TicunaArara	2541	?	?	?	?	?	?	04:11	16:02:01
TicunaArara	2542	24:02:01G	31:01:02G	?	?	07:02:01G	07:02:01G	04:11	04:11
TicunaArara	2543	31:01:02G	31:01:02G	39:03	40:02:01G	03:04:01G	07:02:01G	04:04	08:02:01
TicunaArara	2544	31:01:02G	31:01:02G	39:02:02	39:03	07:02:01G	07:02:01G	04:11	08:04:01
TicunaArara	2545	31:01:02G	31:01:02G	39:03	39:03	07:02:01G	07:02:01G	04:11	08:02:01
TicunaArara	2546	02:13	24:02:01G	15:03:01G	40:02:01G	02:10	03:04:01G	04:11	08:04:01
TicunaArara	2547	24:02:01G	24:02:01G	40:02:01G	52:01:02	03:04:01G	15:02:01G	04:11	16:02:01
TicunaArara	2548	24:02:01G	24:02:01G	40:02:01G	40:02:01G	03:04:01G	03:04:01G	04:04	04:10
TicunaArara	2549	24:02:01G	31:01:02G	35:04:01	39:03	04:01:01G	07:02:01G	08:02:01	08:02:01
TicunaArara	2550	24:02:01G	31:01:02G	?	?	?	?	04:04	04:11
TicunaArara	2551	24:02:01G	24:02:01G	39:09	40:02:01G	03:04:01G	07:02:01G	04:04	08:02:01
TicunaArara	2552	31:01:02G	31:01:02G	39:03	39:03	07:02:01G	07:02:01G	04:11	04:11
TicunaArara	2553	24:02:01G	24:02:01G	39:05	39:05	08:03	08:03	04:11	04:11
TicunaArara	2554	02:13	24:02:01G	35:04:01	35:20:01	04:01:01G	04:01:01G	04:11	08:02:01
TicunaArara	2555	24:02:01G	24:02:01G	15:04	40:02:01G	03:04:01G	04:04:01	?	?
TicunaArara	2557	?	?	35:04:01	52:01:02	?	?	?	?
TicunaArara	2558	02:11:01G	31:01:02G	39:03	52:01:02	07:02:01G	15:02:01G	04:11	16:02:01
TicunaTarapaca	2761	24:02:01G	31:01:02G	35:04:01	39:05	04:01:01G	08:03	04:11	09:01:02
TicunaTarapaca	2762	02:11:01G	24:03:01G	35:06	40:04	03:04:01G	15:02:01G	04:07:01	04:11
TicunaTarapaca	2763	24:02:01G	24:02:01G	35:20:01	35:20:01	?	?	04:04	04:04
TicunaTarapaca	2764	24:02:01G	31:01:02G	35:04:01	35:20:01	04:01:01G	04:01:01G	04:04	04:11
TicunaTarapaca	2765	24:02:01G	24:02:01G	15:04	40:02:01G	01:02:01G	03:04:01G	04:04	08:02:01
TicunaTarapaca	2766	02:11:01G	24:02:01G	40:02:01G	52:01:02	03:04:01G	15:02:01G	04:11	04:11
TicunaTarapaca	2767	24:02:01G	24:02:01G	40:02:01G	40:02:01G	03:04:01G	03:04:01G	04:04	04:11
TicunaTarapaca	2768	24:02:01G	31:01:02G	35:04:01	40:02:01G	03:04:01G	04:01:01G	04:04	04:11
TicunaTarapaca	2769	24:02:01G	24:02:01G	40:02:01G	40:02:01G	03:04:01G	03:04:01G	04:04	04:11
TicunaTarapaca	2770	02:11:01G	31:01:02G	35:04:01	52:01:02	04:01:01G	15:02:01G	04:11	04:11
TicunaTarapaca	2791	24:02:01G	24:02:01G	40:02:01G	40:02:01G	?	?	04:04	04:11

TicunaTarapaca	2792	02:01:01G	24:02:01G	40:02:01G	40:02:01G	03:04:01G	03:04:01G	04:11	04:11
TicunaTarapaca	2793	24:02:01G	31:01:02G	35:04:01	40:02:01G	03:04:01G	04:01:01G	04:11	04:11
TicunaTarapaca	2794	24:03:01G	31:01:02G	40:02:01G	40:02:01G	03:04:01G	03:04:01G	04:04	04:11
TicunaTarapaca	2795	02:11:01G	31:01:02G	35:04:01	52:01:02	04:01:01G	15:02:01G	04:11	04:11
TicunaTarapaca	2796	02:01:01G	24:02:01G	39:05	40:02:01G	03:04:01G	08:03	04:11	09:01:02
TicunaTarapaca	2797	31:01:02G	31:01:02G	39:03	39:03	04:01:01G	07:02:01G	04:04	04:11
TicunaTarapaca	2798	02:01:01G	24:02:01G	40:02:01G	40:02:01G	03:04:01G	03:04:01G	04:04	04:11
TicunaTarapaca	2799	24:02:01G	24:02:01G	15:04	15:04	03:04:01G	03:04:01G	08:02:01	08:02:01
TundraNentsi	2441	02:01:01G	24:02:01G	07:02:01G	44:02:01G	05:01:01G	07:02:01G	01:01:01	15:01:01
TundraNentsi	2442	03:01:01G	32:01:01G	07:02:01G	07:02:01G	07:02:01G	07:02:01G	13:01:01	15:01:01
TundraNentsi	2443	24:02:01G	26:01:01G	07:02:01G	39:01:01G	07:02:01G	12:03:01G	15:01:01	16:01:01
TundraNentsi	2444	02:01:01G	25:01:01	13:02:01	18:01:01G	06:02:01G	12:03:01G	07:01:01	08:01:01G
TundraNentsi	2445	02:01:01G	24:02:01G	39:01:01G	48:01:01G	07:02:01G	08:01:01	04:04	12:01:01G
TundraNentsi	2446	24:02:01G	24:02:01G	07:02:01G	35:01:01G	04:01:01G	07:02:01G	08:01:01G	13:01:01
TundraNentsi	2447	03:01:01G	24:02:01G	44:02:01G	48:01:01G	07:04:01G	08:03	12:01:01G	13:01:01
TundraNentsi	2448	02:01:01G	02:06:01G	35:01:01G	40:02:01G	03:03:01G	03:04:01G	04:01:01	13:01:01
TundraNentsi	2449	02:01:01G	02:01:01G	44:02:01G	48:01:01G	?	?	04:01:01	12:01:01G
TundraNentsi	2450	02:01:01G	02:01:01G	07:02:01G	48:01:01G	07:02:01G	08:01:01	11:01:01/11:01:06	12:01:01G
TundraNentsi	2451	02:01:01G	02:06:01G	48:01:01G	51:01:01G	03:04:01G	08:01:01	12:01:01G	14:03:01
TundraNentsi	2452	02:01:01G	02:06:01G	48:01:01G	51:01:01G	03:04:01G	08:01:01	12:01:01G	14:03:01
TundraNentsi	2453	02:06:01G	02:06:01G	40:02:01G	51:01:01G	03:04:01G	08:01:01	14:03:01	16:02:01
TundraNentsi	2454	02:01:01G	23:01:01G	48:01:01G	49:01	07:01:01G	08:03	12:01:01G	14:01:01G
TundraNentsi	2456	24:02:01G	31:01:02G	27:05:02G	48:01:01G	02:02:02	08:01:01	08:01:01G	12:01:01G
TundraNentsi	2457	02:01:01G	33:01	14:02:01	40:02:01G	03:04:01G	08:02	01:02:01	04:01:01
Waunana	2581	02:11:01G	24:02:01G	39:05	40:04	03:04:01G	07:02:01G	04:07:01	14:02
Waunana	2582	02:01:01G	30:02:01G	18:01:01G	18:01:01G	05:01:01G	05:01:01G	03:01:01	03:01:01
Waunana	2583	02:11:01G	24:02:01G	35:05	40:04	03:04:01G	04:01:01G	04:07:01	04:11
Waunana	2584	02:11:01G	24:03:01G	35:04:01	40:04	03:04:01G	04:01:01G	04:07:01	04:11
Waunana	2585	24:02:01G	24:02:01G	40:02:01G	40:04	03:04:01G	04:01:01G	04:04	14:02
Waunana	2586	24:02:01G	24:02:01G	39:05	40:02:01G	04:01:01G	07:02:01G	04:04	14:02
Waunana	2587	02:11:01G	24:02:01G	15:04	40:04	03:03:01G	03:04:01G	14:02	14:02
Waunana	2588	24:02:01G	24:02:01G	39:05	40:02:01G	04:01:01G	07:02:01G	04:04	14:02
Waunana	2589	24:02:01G	24:02:01G	39:05	40:04	03:04:01G	07:02:01G	14:02	14:02
Waunana	2590	02:11:01G	24:02:01G	15:04	40:02:01G	03:03:01G	04:01:01G	04:04	14:02
Waunana	2591	24:02:01G	24:02:01G	39:05	40:04	03:04:01G	07:02:01G	14:02	14:02
Waunana	2592	24:02:01G	24:02:01G	40:02:01G	52:01:02	03:04:01G	15:02:01G	14:02	16:02:01
Waunana	2593	24:02:01G	24:02:01G	35:10	35:10	03:04:01G	03:04:01G	16:02:01	16:02:01
Waunana	2594	02:11:01G	02:11:01G	15:04	39:05	03:03:01G	07:02:01G	14:02	14:02
Waunana	2595	02:11:01G	24:02:01G	15:04	40:02:01G	03:03:01G	03:04:01G	04:04	14:02
Waunana	2596	02:11:01G	24:02:01G	15:04	35:10	03:03:01G	03:04:01G	14:02	16:02:01
Waunana	2597	02:11:01G	24:02:01G	15:04	40:02:01G	03:03:01G	03:04:01G	04:04	14:02
Waunana	2598	02:13	24:02:01G	15:39	51:01:01G	01:02:01G	15:02:01G	04:04	14:02
Waunana	2599	02:11:01G	24:02:01G	40:02:01G	40:02:01G	03:04:01G	04:01:01G	04:04	14:02
Waunana	2600	02:13	24:02:01G	40:02:01G	51:01:01G	04:01:01G	15:02:01G	04:04	04:04
Wayuu	2522	02:17:01G	24:02:01G	40:02:01G	40:02:01G	03:05	03:05	04:03:01	04:11
Wayuu	2523	02:22:01G	24:02:01G	35:12	35:43:01G	01:02:01G	04:01:01G	04:07:01	16:02:01
Wayuu	2524	24:03:01G	31:02	35:12	51:01:01G	15:02:01G	15:02:01G	04:07:01	14:02
Wayuu	2526	01:01:01G	31:01:02G	08:01:01G	35:11	?	?	14:02	16:02:01
Wayuu	2527	02:13	68:01:02G	40:02:01G	51:01:01G	03:04:01G	15:02:01G	04:04	04:11
Wayuu	2528	02:22:01G	31:01:02G	35:43:01G	40:02:01G	01:02:01G	03:04:01G	04:07:01	04:11
Wayuu	2529	02:01:01G	24:02:01G	35:05	35:12	04:01:01G	04:01:01G	04:03:01	16:02:01
Wayuu	2530	24:02:01G	68:02:01G	18:01:01G	35:12	04:01:01G	14:02:01	04:11	13:02:01
Wayuu	2531	02:01:01G	02:22:01G	27:12	35:49	02:02:02	04:01:01G	08:02:01	15:01:01
Wayuu	2532	24:03:01G	31:01:02G	35:12	39:05	07:02:01G	15:02:01G	04:03:01	16:02:01
Wayuu	2533	25:01:01	30:02:01G	18:01:01G	39:04	07:01:01G	12:03:01G	15:01:01	16:02:01
Wayuu	2534	02:22:01G	31:02	35:49	40:02:01G	03:05	04:01:01G	04:07:01	08:02:01
Wayuu	2535	02:22:01G	30:02:01G	18:01:01G	35:49	04:01:01G	05:01:01G	04:04	08:02:01
Wayuu	2539	24:02:01G	25:01:01	18:01:01G	40:02:01G	03:05	12:03:01G	04:11	15:01:01
Wayuu	2540	24:02:01G	25:01:01	18:01:01G	39:06:01	07:02:01G	12:03:01G	04:04	15:01:01
Zapotec	2081	02:01:01G	24:02:01G	39:02:02	40:27	01:02:01G	03:04:01G	04:04	16:02:01
Zapotec	2082	03:01:01G	03:01:01G	?	?	?	?	01:02:01	04:07:01
Zapotec	2083	02:01:01G	68:01:02G	35:12	40:27	03:04:01G	04:01:01G	04:04	08:02:01
Zapotec	2084	02:01:01G	02:01:01G	35:12	52:01:02	03:03:01G	04:01:01G	14:06	08:02:01
Zapotec	2085	02:01:01G	02:06:01G	35:14:01	39:08	04:01:01G	07:02:01G	16:02:01	04:07:01
Zapotec	2086	02:01:01G	24:02:01G	35:43:01G	39:05	01:02:01G	07:02:01G	04:04	04:07:01
Zapotec	2087	02:06:01G	24:02:01G	35:14:01	35:24	04:01:01G	04:01:01G	16:02:01	14:02

Zapotec	2088	02:06:01G	31:01:02G	15:01:01G-N	15:30	01:02:01G	01:02:01G	14:02	04:03:01
Zapotec	2089	02:01:01G	02:01:01G	35:01:01G	51:01:01G	03:03:01G	03:04:01G	16:02:01	14:06
Zapotec	2090	02:06:01G	31:01:02G	35:14:01	39:02:02	04:01:01G	07:02:01G	16:02:01	14:06
Zapotec	2091	02:06:01G	02:06:01G	?	?	?	?	?	?
Zapotec	2092	02:01:01G	02:01:01G	47:01:01G	47:01:01G	?	?	?	?
Zapotec	2093	02:06:01G	02:06:01G	15:30	39:05	01:02:01G	07:02:01G	04:04	04:07:01
Zapotec	2094	24:02:01G	32:01:01G	39:05	53:01:01	04:01:01G	07:02:01G	13:02:01	08:02:01
Zapotec	2095	02:06:01G	02:06:01G	?	?	?	?	04:03:01	04:04
Zapotec	2096	02:01:01G	68:03:01	39:05	47:01:01G	07:01:01G	07:02:01G	04:05:01/04:05:03	04:07:01
Zapotec	2097	02:06:01G	31:01:02G	35:17	39:02:02	03:04:01G	04:01:01G	04:04	08:02:01
Zapotec	2098	02:01:01G	02:06:01G	39:02:02	52:01:02	03:03:01G	07:02:01G	16:02:01	08:02:01
Zapotec	2099	02:01:01G	02:01:01G	15:17	35:12	04:01:01G	07:01:01G	13:02:01	08:02:01
Zapotec	2100	02:01:01G	02:01:01G	35:17	58:01:01G	04:01:01G	07:01:01G	10:01:01	04:10
Zenu	2482	02:06:01G	02:22:01G	39:05	40:02:01G	03:04:01G	03:05	04:07:01	16:02:01
Zenu	2484	24:02:01G	31:01:02G	35:43:01G	35:49	01:02:01G	04:01:01G	03:02:02	14:06
Zenu	2485	?	?	35:49	35:49	?	?	?	?
Zenu	2486	?	?	07:02:01G	40:02:01G	?	?	?	?
Zenu	2489	24:02:01G	68:01:02G	35:43:01G	51:10	01:02:01G	15:02:01G	03:02:02	03:02:02
Zenu	2490	?	?	35:49	35:43:01G	?	?	?	?
Zenu	2491	24:02:01G	24:03:01G	35:43:01G	40:02:01G	01:02:01G	03:04:01G	03:02:02	03:02:02
Zenu	2492	24:02:01G	31:01:02G	35:12	35:43:01G	01:02:01G	15:02:01G	03:02:02	14:02
Zenu	2493	24:02:01G	24:02:01G	40:02:01G	40:02:01G	03:04:01G	03:04:01G	03:02:02	03:02:02
Zenu	2494	?	?	40:02:01G	51:10	?	?	?	?
Zenu	2495	02:22:01G	68:01:02G	35:49	51:10	04:01:01G	15:02:01G	03:02:02	03:02:02
Zenu	2496	02:22:01G	68:01:02G	35:01:01G	51:10	04:01:01G	15:02:01G	03:02:02	04:05:04
Zenu	2497	02:06:01G	24:02:01G	39:19:02	40:02:01G	03:05	07:02:01G	04:07:01	16:02:01
Zenu	2498	24:02:01G	68:01:02G	40:02:01G	51:10	03:04:01G	15:02:01G	03:02:02	03:02:02
Zenu	2499	02:22:01G	24:02:01G	35:01:01G	35:43:01G	01:02:01G	04:01:01G	03:02:02	04:07:01
Zenu	2500	24:02:01G	68:01:02G	18:01:01G	51:10	12:03:01G	15:02:01G	03:02:02	11:04:01
