# Supplementary material for: Demographic history and selection at HLA loci in Native Americans
Source: PLoS One. 2020 Nov 4;15(11):e0241282. doi: 10.1371/journal.pone.0241282 (PMC7641399; doi:10.1371/journal.pone.0241282)
Supplement: S2 Table — (PDF) [file pone.0241282.s002.pdf]

S2 Table details:

The HLA typing in the studies from the Solberg et al. (2008) meta-analysis was carried out over an extended period of time. Some of the typing methods used in earlier studies may not have been able to unambiguously distinguish among certain sets of alleles that can be distinguished using more recent typing methods. This type of naming inconsistency could lead to the possibility of an allele that we classify as endemic actually being shared among regions (but given different names across studies). We omitted migrant populations with a complexity level of three (labeled as “3mig”) and populations whose continental origin classification was “other” from the Solberg et al. (2008) dataset.

In order to determine the set of alleles in the present study that could have been called by a different name in earlier studies (i.e., studies included in the Solberg et al. (2008) dataset), we developed a disambiguity score based on NMDP codes for sets of alleles that may not be distinguished unambiguously. For example, *A*01:01/01:02/01:03* and *A*01:01/01:04/01:05/01:22* are 2 different allele sets for HLA-A. In the first set, *A*01:01* cannot be distinguished from *A*01:02* or *A*01:03*. In the second set *A*01:01* can be distinguished from *A*01:02* or *A*01:03*, but cannot be distinguished from and *A*01:04, A*01:05, or A*01:22*. The disambiguity score was defined for each pair of alleles and takes a value of 0 if the two alleles are never disambiguated (distinguished) and a value of 1 if they are always disambiguated. The score is a frequency based measure that is based on the fraction of times that any two given alleles occur in an ambiguous set in the NMDP database of over millions of typings.

The above disambiguity score was used in the following way. For each endemic or LFD allele, we identified those whose pairwise disambiguity score with the LFD/Endemic allele was below a threshold value. For these "pairwise-ambiguous" alleles, we checked their frequency counts in different world regions. These pairwise-ambiguous alleles are noted in Supplementary Table S1.

Table S2. Disambiguity score for HLA alleles.

Locus	Endemic Allele	Alternate allele	Disambig. Score	Designation	Avg Frequency among populations sharing allele: Solberg et al. non-Amerindian	Solberg et al. Amerindian	Present Study Amerindian
**DRB1**	**08:07**	-	-	Endemic	-	0.0900	0.0830
		08:02	0.320	LFD	0.0246	0.1600	0.1650
		08:04	0.439		0.0178	0.0290	0.0450
		08:11	0.010	LFD	0.0071	0.0930	0.1140
**A**	**02:64**	-	-	Endemic	-	0.0240	0.0240
		02:01	0.519		0.1348	0.2570	0.2400
		02:06	0.513	LFD	0.0546	0.1890	0.2170
		02:07	0.364		0.0511	0.0240	0.0240
		02:20	0.359		0.0050		
		02:24	0.369		0.0040		
		02:25	0.192		0.0045		
		02:26	0.358		0.0029		
		02:30	0.357		0.0051		
	**31:15**	-	-	Endemic	-	0.0500	0.0500
	**68:05**	-	-	Endemic	-	0.0140	0.0260
		68:12	0.513		0.0071		
	**68:16**	-	-	Endemic	-	0.0250	0.0250
	**68:17**	-	-	Endemic	-	0.0750	0.1000
		68:01	0.465	LFD	0.0334	0.1280	0.1380
		68:03	0.570	LFD	0.0056	0.0380	0.0580
		68:23	0.520	Endemic	-	0.0250	0.0250
	**68:23**	-	-	Endemic	-	0.0250	0.0250
		68:01	0.590	LFD	0.0334	0.1280	0.1380
		68:03	0.227	LFD	0.0056	0.0380	0.0580
		68:17	0.520	Endemic	-	0.0750	0.1000
	**68:30**	-	-	Endemic	-	0.0790	0.0790
		68:06	0.232		0.0111		
	**68:47**	-	-	Endemic	-	0.0250	0.0250
**C**	**02:07**	-	NA	Endemic	-	0.0260	0.0260
	**03:57**	-	NA	Endemic	-	0.0250	0.0250
	**06:16**	-	NA	Endemic	-	0.0280	0.0280
	**15:09**	-	NA	Endemic	-	0.0250	0.0250

	**15:10**	-	NA	Endemic	-	0.0360	0.0360
**B**	**15:04**	-	-	Endemic	-	0.1250	0.1560
		15:39	0.435		0.0030	0.0250	0.0250
	**15:30**	-	-	Endemic	-	0.0360	0.0590
	**35:19**	-	-	Endemic	-	0.1040	0.0500
		35:04	0.571	LFD	0.0021	0.0560	0.0770
		35:06	0.459	LFD	0.0164	0.0540	0.0260
		35:11	0.459	LFD	0.0040	0.0370	0.0330
		35:12	0.469	LFD	0.0100	0.0920	0.1070
		35:14	0.513	LFD	0.0039	0.0630	0.0630
		35:17	0.528	LFD	0.0079	0.0440	0.0460
		35:20	0.475	LFD	0.0162	0.0340	0.0570
		35:21	0.470	LFD	0.0055	0.0330	0.0500
		35:23	0.409	Endemic	-	0.0690	0.1000
		35:24	0.430	Endemic	-	0.0190	0.0290
		35:43	0.459	LFD	0.0048	0.1280	0.2000
		35:49	0.358	Endemic	-	0.0950	0.0950
		35:13	0.414		0.0090		
		35:16	0.535		0.0200	0.0030	
		35:25	0.515		0.0600		
		35:27	0.515		0.0404		
		35:28	0.548		0.0042		
		35:32	0.438		0.0100		
	**35:23**	-	-	Endemic	-	0.0690	0.1000
		35:04	0.440	LFD	0.0021	0.0560	0.0770
		35:06	0.238	LFD	0.0164	0.0540	0.0260
		35:10	0.513	LFD	0.0073	0.0570	0.0920
		35:11	0.273	LFD	0.0040	0.0370	0.0330
		35:12	0.255	LFD	0.0100	0.0920	0.1070
		35:14	0.341	LFD	0.0039	0.0630	0.0630
		35:17	0.361	LFD	0.0079	0.0440	0.0460
		35:19	0.493	Endemic	-	0.1040	0.0500
		35:20	0.259	LFD	0.0162	0.0340	0.0570
		35:21	0.442	LFD	0.0055	0.0330	0.0500
		35:24	0.459	Endemic	-	0.0190	0.0290
		35:43	0.350	LFD	0.0048	0.1280	0.2000
		35:49	0.229	Endemic	-	0.0950	0.0950
		35:13	0.467		0.0090		
		35:15	0.156		0.0054		
		35:16	0.338		0.0200	0.0030	
		35:25	0.336		0.0600		
		35:28	0.527		0.0042		
		35:32	0.400		0.0100		
		35:34	0.204		0.0035		

**35:24**	-	-	Endemic	-	0.0190	0.0290
	35:04	0.582	LFD	0.0021	0.0560	0.0770
	35:06	0.477	LFD	0.0164	0.0540	0.0260
	35:10	0.519	LFD	0.0073	0.0570	0.0920
	35:11	0.394	LFD	0.0040	0.0370	0.0330
	35:12	0.487	LFD	0.0100	0.0920	0.1070
	35:14	0.522	LFD	0.0039	0.0630	0.0630
	35:17	0.542	LFD	0.0079	0.0440	0.0460
	35:19	0.493	Endemic	-	0.1040	0.0500
	35:20	0.595	LFD	0.0162	0.0340	0.0570
	35:21	0.358	LFD	0.0055	0.0330	0.0500
	35:23	0.303	Endemic	-	0.0690	0.1000
	35:43	0.356	LFD	0.0048	0.1280	0.2000
	35:49	0.467	Endemic	-	0.0950	0.0950
	35:13	0.442		0.0090		
	35:16	0.524		0.0200	0.0030	
	35:25	0.530		0.0600		
	35:28	0.561		0.0042		
	35:32	0.463		0.0100		
**35:48**	-	-	Endemic	-	0.0260	0.0260
**35:49**	-	-	Endemic	-	0.0950	0.0950
	35:04	0.426	LFD	0.0021	0.0560	0.0770
	35:06	0.200	LFD	0.0164	0.0540	0.0260
	35:10	0.509	LFD	0.0073	0.0570	0.0920
	35:11	0.254	LFD	0.0040	0.0370	0.0330
	35:12	0.223	LFD	0.0100	0.0920	0.1070
	35:14	0.314	LFD	0.0039	0.0630	0.0630
	35:17	0.342	LFD	0.0079	0.0440	0.0460
	35:19	0.498	Endemic	-	0.1040	0.0500
	35:20	0.236	LFD	0.0162	0.0340	0.0570
	35:21	0.448	LFD	0.0055	0.0330	0.0500
	35:23	0.438	Endemic	-	0.0690	0.1000
	35:24	0.341	Endemic	-	0.0190	0.0290
	35:43	0.196	LFD	0.0048	0.1280	0.2000
	35:13	0.204		0.0090		
	35:15	0.463		0.0054		
	35:16	0.066		0.0200	0.0030	
	35:25	0.594		0.0600		
	35:27	0.317		0.0404		
	35:28	0.319		0.0042		
	35:32	0.527		0.0100		
	35:34	0.381		0.0035		
**35:99**	-	-	Endemic	-	0.1760	0.1760
**35:102**	-	-	Endemic	-	0.0280	0.0280
**39:08**	-	-	Endemic	-	0.0220	0.0280

	39:02	0.036	LFD	0.0045	0.1210	0.1160
	39:04	0.416		0.0022	0.0220	0.0330
	39:11	0.225	Endemic	-	0.0290	0.0710
	39:10	0.534		0.0091		
	39:12	0.123		-	0.0430	
	39:13	0.016		-	0.0060	
	39:15	0.317		0.0031		
**39:11**	-	-	Endemic	-	0.0290	0.0710
	39:02	0.540	LFD	0.0045	0.1210	0.1160
	39:05	0.420	LFD	0.0038	0.0920	0.0940
	39:08	0.534	Endemic	-	0.0220	0.0280
	39:09	0.473	LFD	0.0114	0.1220	0.1620
	39:10	0.565		0.0091		
	39:12	0.559		-	0.0430	
	39:13	0.536		-	0.0060	
	39:15	0.565		0.0031		
**39:19**	-	-	Endemic	-	0.0310	0.0310
	39:03	0.526	LFD	0.0363	0.0790	0.1690
	39:04	0.596		0.0022	0.0220	0.0330
**40:27**	-	-	Endemic	-	0.0590	0.0590
	40:04	0.558	LFD	0.0010	0.1190	0.1770
**40:64**	-	-	Endemic	-	0.0240	0.0240
**51:13**	-	-	Endemic	-	0.0260	0.0260
	51:08	0.591	LFD	0.0067	0.0260	0.0470
	51:02	0.515	LFD	0.0101	0.0520	
	51:04	0.244	LFD	0.0041	0.0470	
	51:05	0.468		0.0037	0.0060	
	51:06	0.325		0.0112	0.0110	
	51:07	0.273		0.0104		
	51:09	0.506		0.0023		
	51:21	0.239		0.0008		
	51:22	0.114		0.0050		
	51:34	0.123		0.0008		
